# A phosphorylation switch controls the spatiotemporal activation of Rho GTPases in directional cell migration

**DOI:** 10.1038/ncomms8721

**Published:** 2015-07-13

**Authors:** Xuan Cao, Tomonori Kaneko, Jenny S. Li, An-Dong Liu, Courtney Voss, Shawn S. C. Li

**Affiliations:** 1 Department of Biochemistry, Schulich School of Medicine and Dentistry, Western University, London, Ontario, Canada N6A 5C1; 2 Department of Medical Genetics, School of Basic Medicine, Tongji Medical College, Huazhong University of Science and Technology, Wuhan, 430030, China; 3 Department of Medicine, Schulich School of Medicine and Dentistry, Western University, London, Ontario, Canada N6A 5C1; 4 Children's Health Research Institute, 800 Commissioners Road East, London, Ontario, Canada N6C 2V5

## Abstract

Although cell migration plays a central role in development and disease, the underlying molecular mechanism is not fully understood. Here we report that a phosphorylation-mediated molecular switch comprising deleted in liver cancer 1 (DLC1), tensin-3 (TNS3), phosphatase and tensin homologue (PTEN) and phosphoinositide-3-kinase (PI3K) controls the spatiotemporal activation of the small GTPases, Rac1 and RhoA, thereby initiating directional cell migration induced by growth factors. On epidermal growth factor (EGF) or platelet-derived growth factor (PDGF) stimulation, TNS3 and PTEN are phosphorylated at specific Thr residues, which trigger the rearrangement of the TNS3–DLC1 and PTEN–PI3K complexes into the TNS3–PI3K and PTEN–DLC1 complexes. Subsequently, the TNS3–PI3K complex translocates to the leading edge of a migrating cell to promote Rac1 activation, whereas PTEN–DLC1 translocates to the posterior for localized RhoA activation. Our work identifies a core signalling mechanism by which an external motility stimulus is coupled to the spatiotemporal activation of Rac1 and RhoA to drive directional cell migration.

## Abstract (web summary)

Directed cell migration requires spatially regulated activity of GTPases Rac1 and RhoA. Here Cao *et al*. show that growth factor stimulation promotes phosphorylation of tensin-3 and phosphatase and tensin homologue (PTEN) and their association with PI 3-kinase and deleted in liver cancer 1 (DLC1) to regulate GTPase activity.

## Graphical abstract

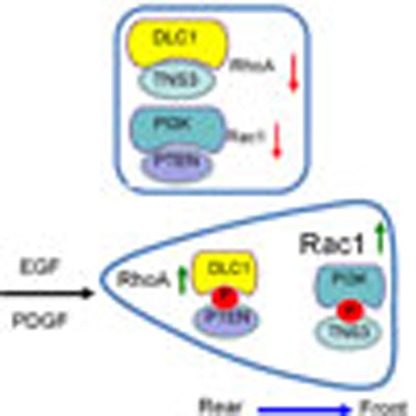

Directional cell migration plays a critical role in embryonic development, immune surveillance and wound healing^1^. Uncontrolled cell migration, on the other hand, is associated with a host of conditions, notably cancer metastasis^2^. Cell migration is orchestrated by numerous regulators which, together, control the dynamic changes in the plasma membrane and cytoskeleton^3^. The Rho family of small GTPases, to which RhoA, Rac1 and Cdc42 belong, plays an essential role in cell migration by promoting cytoskeletal reorganization necessary for motility^4^,^5^,^6^. While the activation of Rac1 and Cdc42 leads to the formation of lamellipodia and filopodia, respectively, active RhoA is required for the formation of actin stress fibres and focal adhesions^5^,^7^. A small GTPase cycles between the GTP-bound (active) and GDP-bound (inactive) states, with their interconversion catalysed by guanine nucleotide exchange factors (GEFs), which promotes GDP exchange for GTP; and by GTPase-activating proteins (GAPs), which speed up GTP hydrolysis^6^,^8^,^9^. Moreover, the GDP dissociation inhibitors may also affect the activity of the Rho GTPases^10^.

The tumour suppressor DLC1 plays an important role in cell migration and transformation by virtue of its GAP activity towards RhoA^11^,^12^. DLC1, which features a SAM (sterile alpha motif), RhoGAP and START (STAR-related lipid transfer) domain, promotes the hydrolysis of GTP-bound RhoA and, to a lesser extent, Cdc42, but not Rac1 (refs 13, 14, 15). The tumour suppressive activity of DLC1 requires the RhoGAP domain and its binding to tensins, a family of focal adhesion proteins including Tensin-1, -2, -3, and -4 (or Cten)^15^,^16^. Except for Cten, all tensin proteins contain an actin-binding domain (ABD)^17^. We have shown recently that TNS3 markedly enhances the RhoGAP activity of DLC1 by converting the latter from an auto-inhibited to an active state^18^.

To initiate directional cell migration, Rac1 is activated at the leading edge of a migrating cell where it promotes the formation of lamellipodial protrusions^19^. RhoA, in contrast, is activated in the posterior and/or lateral region where it facilitates the generation of contractile force to propel the cell forward^7^,^20^,^21^. How this polarized pattern of active Rac1 and RhoA is established to drive cell migration is not fully understood. Studies with neutrophils and *D. discoideum* have shown that PI3K and PIP3 play a pivotal role in directional sensing during chemotaxis^22^,^23^. PI3K catalyses the conversion of phosphatidylinositol (4,5)-bisphosphate to phosphatidylinositol (3,4,5)-trisphosphate (PIP3), the latter of which is recognized by pleckstrin homology (PH) domains. PI3K is composed of a catalytic (p110) and a regulatory (p85) subunit, each of which has multiple isoforms. It has been shown that PIP3 promotes Rac1 activation by recruiting Tiam1, a PH domain-containing GEF, to the leading edge of migrating cells^24^,^25^.

While PI3K promotes membrane protrusion via PIP3, the tumour suppressor PTEN inhibits this process^26^,^27^. PTEN harbours a phosphatase domain that can convert PIP3 back to phosphatidylinositol (4,5)-bisphosphate and a C2 domain known to bind phospholipids^28^. Contrary to PI3K and PIP3 that localize to membrane protrusions, PTEN is found mainly in the posterior of migrating *Dictyostelium*, mouse neutrophils or human embryonic kidney (HEK) 293 cells^29^,^30^,^31^.

Directional migration may be triggered by external guidance cues or mediated by intrinsic cell directionality^1^. Directional migration induced by growth factors such as EGF, PDGF and fibroblast growth factor belongs to the latter^32^,^33^,^34^. To initiate wound healing, a number of growth factors and cytokines are released to stimulate epithelial cell migration, among other effects^34^. How do growth factors regulate cell motility? We address this fundamental question by delineating the molecular mechanism governing growth factor-induced epithelial cell migration. We find that EGF signalling leads to the phosphorylation of TNS3 and PTEN, which, in turn, prompts a switch of binding partners for TNS3 and PTEN such that the TNS3–DLC1 and PTEN–PI3K complexes are dissolved to form the TNS3–PI3K and PTEN–DLC1 complexes. The latter complexes then mobilize to specific subcellular locations to promote spatially restricted activation of Rac1 and RhoA conducive to migration. Therefore, a ‘phosphorylation switch' (P-switch) involving TNS3, PTEN, DLC1 and PI3K links an external signal (for example, EGF) to the spatiotemporal activation of the Rho GTPases to enable directional cell migration.

## Results

### EGF regulates TNS3 or PTEN binding to DLC1 or PI3K

**Figure 1:**
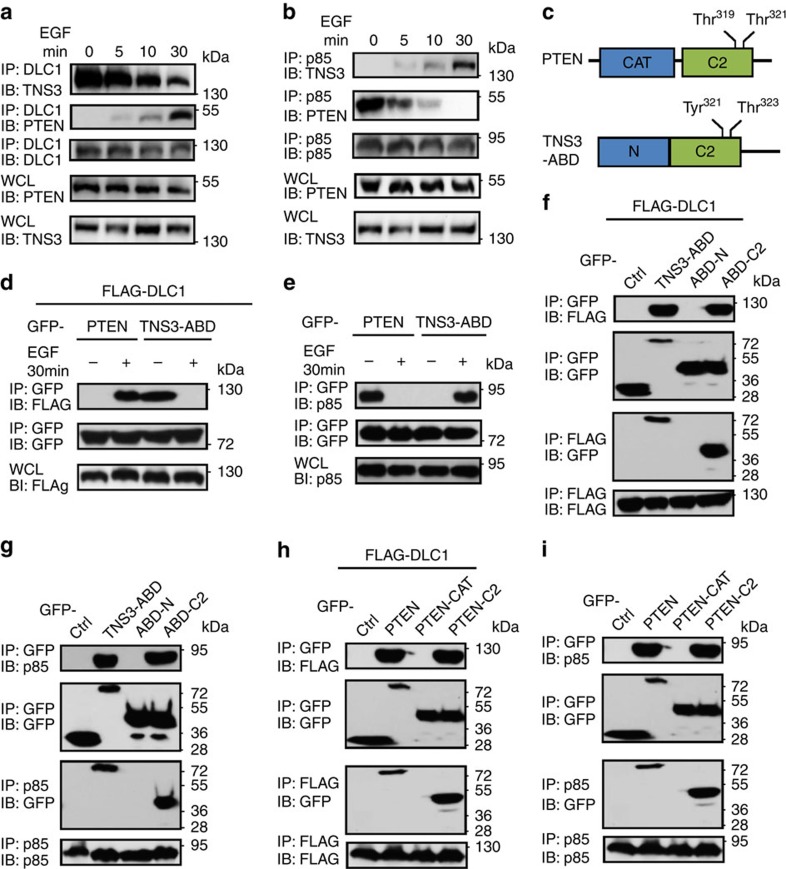
EGF regulates the dynamic interactions of DLC1, TNS3, PTEN and PI3K. (**a**) Acute EGF treatment promoted PTEN, but diminished TNS3 binding to DLC1. MCF10A cells were serum starved and incubated with EGF or not for 5, 15 or 30 min before IP and IB with the specified antibodies. WCL, whole-cell lysate. (**b**) Acute EGF treatment gradually promoted PI3K binding to TNS3 at the expense of PTEN. MCF10A cells under the same conditions as in **a** were subjected to IP/IB using the indicated antibodies. An anti-p85 antibody was used to detect PI3K. (**c**) The domain structures of PTEN and TNS3–ABD. The two Thr phosphorylation sites in the PTEN-C2 domain and the equivalent residues in the ABD C2 domain are identified. (**d**) EGF treatment of HEK293 cells enhanced PTEN, but abolished TNS3–ABD binding to DLC1. PTEN and TNS3–ABD were expressed as GFP fusions and DLC1 with a FLAG-tag to facilitate IP/IB analysis. (**e**) A PTEN–PI3K(p85) complex was replaced by a TNS3(ABD)–PI3K(p85) complex in response to EGF treatment in HEK293 cells overexpressing these proteins. (**f**) The C2 domain, but not the amino-terminal region of TNS3–ABD, mediated binding to DLC1. HEK293 cells co-expressing FLAG-DLC1 and GFP fusion TNS3–ABD, ABD-N or the -C2 domain were subjected to IP/IB analysis using anti-GFP and anti-FLAG antibodies. (**g**) The C2 domain was responsible for TNS3–ABD binding to PI3K–p85. GFP-ABD, -ABD-N or -C2 was examined for binding to endogenous p85 in HEK293 cells. (**h**) The C2, but not the catalytic domain (CAT), mediated binding of PTEN to DLC1. Full-length PTEN, or its catalytic or C2 domain was expressed as GFP fusion and examined for binding to FLAG-DLC1 in HEK293 cells. (**i**) The PTEN-C2, but not the catalytic domain, bound to endogenous p85 in HEK293 cells. Cells used in **f**–**i** were cultured in serum-containing media. Data shown are representative of three independent experiments.

We employed the human MCF10A mammary epithelial cells to investigate the mechanism of cell migration. Acute EGF treatment (≤30 min) following serum starvation triggered cell scattering and directional migration without eliciting significant changes in protein level for DLC1, TNS3, PI3K and PTEN (Supplementary Fig. 1). To find out if EGF would affect the interactions of these cell motility regulators with each other^18^,^24^,^27^,^35^, we stimulated serum-starved MCF10A cells with EGF for 5, 10 or 30 min. Endogenous protein–protein interactions were then monitored by immunoprecipitation (IP) followed by immunoblotting (IB) using appropriate antibodies. TNS3 bound strongly to DLC1 before EGF treatment^18^. However, the TNS3–DLC1 interaction started to weaken at 10 min of EGF stimulation and was markedly attenuated at 30 min (Fig. 1a). In contrast, PTEN, which has been previously shown to bind DLC1 (ref. 36), exhibited no binding to the latter in the absence of EGF; yet the two proteins bound to each other progressively in response to EGF stimulation (Fig. 1a). A similar trend was observed for the TNS3–PI3K (via the p85 subunit) interaction as it became stronger on EGF treatment. At the same time, the PTEN–p85 interaction decreased gradually with EGF treatment and was completely lost at 30 min (Fig. 1b). Therefore, EGF stimulation led to a swap of binding partners for TNS3 and PTEN such that the TNS3–DLC1 and PTEN–PI3K complexes were replaced by the TNS3–PI3K and PTEN–DLC1 complexes. This binding-partner switch was evident at 5 min, which became substantial at 10 min, and complete at 30 min of EGF stimulation.

### C2 domain mediates binding of TNS3 or PTEN to DLC1 or PI3K

Because the ABD exhibits sequence identity to PTEN^37^ (Fig. 1c and Supplementary Fig. 2), we interrogated whether it mediated TNS3 binding to DLC1 or PI3K and found that the TNS3–ABD was indeed capable of binding to DLC1. Similar to the full-length TNS3, the ABD–DLC1 interaction was abolished by acute EGF treatment in HEK293 cells (Fig. 1d) and, the ABD associated with PI3K–p85 in the presence, but not in the absence of EGF (Fig. 1e). Moreover, a green fluorescent protein (GFP)–PTEN fusion protein expressed in HEK293 underwent the binding-partner switch from PI3K to DLC1 in response to EGF stimulation (Fig. 1d,e and Supplementary Fig. 3).

The TNS3–ABD contains a putative C2 domain homologous to the PTEN-C2 domain^37^ (Fig. 1c and Supplementary Fig. 2). Because the latter plays a role in cell migration^38^, we wondered if the C2 domain might mediate dynamic binding of PTEN or TNS3–ABD to DLC1 or PI3K. TNS3–ABD, PTEN or a truncation mutant (Supplementary Fig. 4) was expressed as GFP fusion in HEK293 together with FLAG-DLC1. Reciprocal IP/IB experiments showed that the C2 domain, but not the amino-terminal region of ABD (ABD-N), mediated binding of the latter to either FLAG-DLC1 (Fig. 1f) or endogenous PI3K (Fig. 1g). Similarly, the C2, and not the catalytic domain, was responsible for PTEN binding to either DLC1 or PI3K (Fig. 1h,i).

### The C2 domain is phosphorylated following MAPK activation

**Figure 2:**
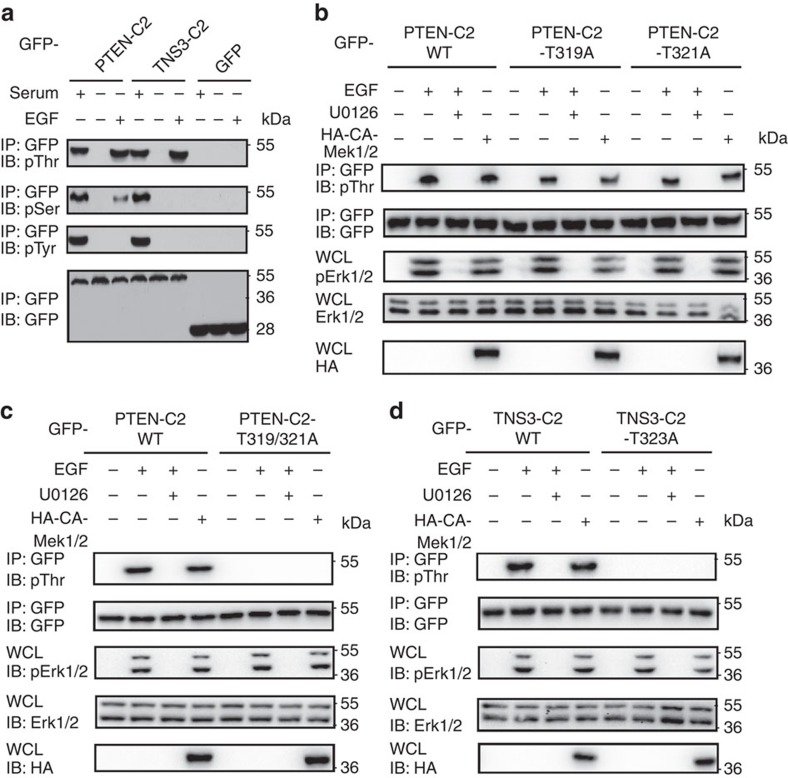
C2 domains were phosphorylated on specific Thr residues. (**a**) The PTEN and TNS3 C2 domains were phosphorylated. The C2 domains were expressed in HEK293 cells and examined for Ser, Thr or Tyr phosphorylation using specific antibodies following EGF treatment for 30 min. GFP was included as a control. (**b**) The Mek1/2 kinase is involved in phosphorylating the PTEN-C2 domain. HEK293 cells expressing the GFP-PTEN-C2 domain, or the T319A or T321A mutant, were co-transfected with a construct for HA-CA-Mek1/2 or treated with EGF or U0126, a Mek1/2 inhibitor. Protein phosphorylation was determined using an anti-pThr antibody. Whole-cell lysates (WCL) were IB for total Erk1/2, phosphorylated Erk1/2 (pErk1/2) or HA. (**c**) The PTEN-C2 domain double mutant, T319/321A was no longer Thr phosphorylated following EGF stimulation or CA-Mek1/2 overexpression. (**d**) The TNS3-C2 domain, but not the T323A mutant, was phosphorylated following Mek1/2 overexpression or EGF stimulation. Data shown are representative of three independent experiments.

The PTEN-C2 domain harbours four potential Ser/Thr phosphorylation sites^31^, one of which is conserved in the TNS3-C2 domain (Supplementary Fig. 2). Indeed, robust Thr phosphorylation was observed on both C2 domains following EGF stimulation of serum-starved HEK293 cells (Fig. 2a). Because EGF signalling activates the MAPK pathway, which then leads to Mek1/2 and Erk1/2 activation^39^, we investigated if the C2 domain could be phosphorylated by the former, taking advantage of an HA-tagged, constitutively active mutant of Mek1/2, HA-CA-Mek1/2 (refs 40, 41). CA-Mek1/2 promoted, whereas U0126, a Mek1/2 inhibitor, blocked both the C2 domain phosphorylation (Fig. 2b,d), suggesting that Mek1/2 or Erk1/2 is involved in phosphorylating the C2 domains. Because TNS3 could also be phosphorylated on Tyr residues by Src^42^, we tested if this event would regulate the binding specificity of TNS3. Although the application of the Src-specific inhibitor I (Src-I1) caused a marked reduction in TNS3 Tyr phosphorylation induced by EGF, it had no effect on TNS3 binding to either DLC1 or PI3K (Supplementary Fig. 5). This indicates that Tyr phosphorylation does not play a role in the dynamic interactions of TNS3 with DLC1 or PI3K.

To identify the site(s) of phosphorylation, we created mutants of the PTEN-C2 domain in which each potential phosphorylation site was replaced by an Ala. The resulting mutants S229A and T232A showed no difference from the wild-type (wt) C2 domain in binding to DLC1 or PI3K (Supplementary Fig. 6), suggesting either that these residues are not phosphorylated or that their phosphorylation is unimportant for binding. We subsequently transfected HEK293 cells with an expression construct for either the T319A or T321A single-mutant or the T319/321A double mutant. While the phosphorylation levels of the single mutants were reduced compared with the wt C2 domain, the double mutant was no longer Thr phosphorylated in response to EGF stimulation or following CA-Mek1/2 expression (Fig. 2b,c). This indicates that both Thr319 and Thr321in the PTEN-C2 domain were phosphorylated following MAPK activation. Thr323 in the TNS3-C2 domain, equivalent to Thr321 in PTEN-C2 (Fig. 1d), was identified as the sole phosphorylation site as its substitution by an Ala abolished phosphorylation of the corresponding mutant, TNS3-C2-T323A (Fig. 2d).

Phosphorylation of a carboxyl-terminal Ser/Thr-rich motif (^380^SDTTDS^385^) in PTEN has been shown to regulate its membrane association^43^. To find out if this motif is phosphorylated and/or involved in binding DLC1 or PI3K–p85, we generated PTEN (1–350) that contains the C2 domain but missing the carboxyl-terminal end, and PTEN (351–403) that harbours the Ser/Thr-rich motif but not the C2 domain. The two fragments were expressed, respectively, in HEK293 cells and examined for phosphorylation by Mek1/2 and binding to DLC1 or PI3K. As shown in Supplementary Fig. 7, PTEN (351–403) was neither Ser/Thr phosphorylated by Mek1/2, nor was it involved in binding DLC1 or p85.

### A P-switch for dynamic protein interactions

**Figure 3:**
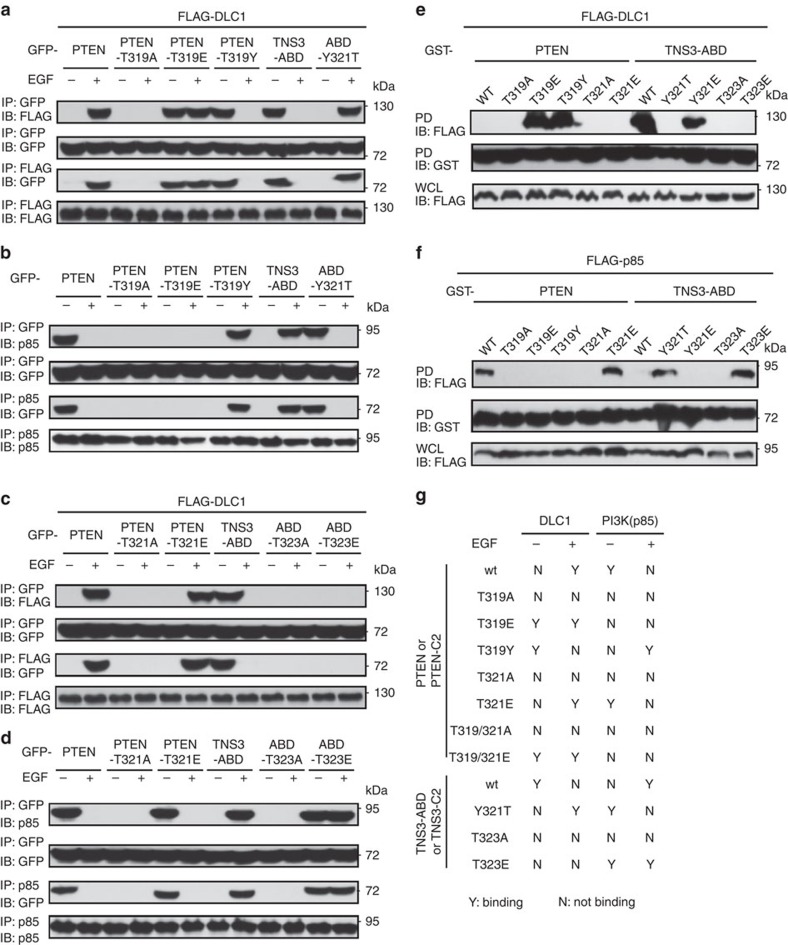
Two equivalent residues in TNS3 and PTEN dictate the binding specificity. (**a**) Thr319 in PTEN, and the equivalent Tyr321 in TNS3–ABD, controlled their dynamic interactions with DLC1 in response to EGF treatment. HEK293 cells co-expressing DLC1 and wt PTEN or a mutant (T319A, T319E or T319Y), or wt TNS3–ABD or the Y321T mutant were subjected to IP/IB to examine their interactions in the absence (−) or presence (+) of EGF stimulation. DLC1 was tagged with FLAG; PTEN, TNS3 or the mutants were fused to GFP. (**b**) The same panel of proteins as in **a** were examined for binding to endogenous PI3K (via p85). (**c**,**d**) Thr321 in PTEN and Thr323 in TNS3–ABD play an important role in dictating binding to DLC1 (**c**) or PI3K–p85 (**d**). (**e**,**f**) Recombinant GST–PTEN, GST–TNS3–ABD or an indicated mutant was used to pull down FLAG-DLC1 (**e**) or FLAG-p85 (**f**) from HEK293 cells. The bound proteins were detected by anti-FLAG IBs. (**g**) A summary of binding specificities displayed by the panel of mutants used in **a**–**f** and in Supplementary Fig 6 and 8. Data shown are representative of three independent experiments.

Phosphorylation of the C2 domain following EGF stimulation provides a plausible mechanism by which to regulate the dynamic interactions of TNS3 and PTEN with DLC1 and PI3K. We investigated this possibility employing mutants of PTEN and TNS3 or of the corresponding C2 domain in which a Thr phosphorylation site was replaced by an Ala or a Glu residue or swapped with the equivalent residue in the other C2 domain. While the PTEN/C2-T319A mutants failed to bind DLC1, the T319E mutants bound DLC1 constitutively (Fig. 3a and Supplementary Fig. 8a), indicating that Thr319 phosphorylation is necessary for the PTEN/C2-DLC1 interaction. Remarkably, the PTEN/C2-T319Y mutants, in which Thr319 was swapped with a Tyr (Y) residue at the equivalent position in TNS3 (Fig. 1c), behaved like wt TNS3-ABD or TNS3-C2. Conversely, replacing Tyr321 in the TNS3–ABD/C2 with the equivalent Thr residue in PTEN yielded mutants that behaved like the wt PTEN or PTEN-C2 domain in binding to DLC1 (Fig. 3a and Supplementary Fig. 8a). Binding specificity switch was also observed for the same mutants to PI3K (Fig. 3b and Supplementary Fig. 8b).Therefore, a single residue, Thr319 in PTEN or Tyr321 in TNS3, dictates the binding specificity of PTEN and TNS3 to DLC1 or PI3K in response to EGF signalling.

In contrast to the specificity-determining role of Thr319, the function of Thr321 in the PTEN–PI3K/TNS3 interactions is less clear. While the PTEN/C2-T321A mutants failed to bind DLC1, the T321E mutants exhibited characteristics of the wt protein/C2 domain (Fig. 3c and Supplementary Fig. 8c). Similar results were obtained for the above mutants in binding PI3K (Fig. 3d and Supplementary Fig. 8d). Moreover, the double mutants PTEN/C2-T319/321A and -T319/321E behaved like the single mutants T319A and T319E in binding to DLC1 (Supplementary Fig. 8e,f). It thus appears that the specificity and affinity of the PTEN-C2 domain are jointly controlled by both Thr319 and Thr321, with the latter playing a ‘fine-tuning' role. Notwithstanding this assertion, substitution of Thr321 by Ala abolished PTEN/C2 binding to both DLC1 and PI3K–p85, suggesting that the side chain of Thr321 is directly involved in ligand binding. The equivalent TNS3–ABD/C2 mutants behaved differently-whereas the ABD/C2-T323A mutants failed to bind DLC1 and PI3K, the T323E mutants bound PI3K constitutively, but failed to bind DLC1 (Fig. 3c,d and Supplementary Fig. 8c,d). Similar results were obtained when the mutations were introduced to TNS3 (Supplementary Fig. 9). The only difference was that a basal level binding between DLC1 and TNS3 or a mutant was evident due probably to the presence of an SH2 and a PTB domain in TNS3 that may mediate binding to DLC1 in a phosphorylation-independent manner^44^.

To rule out the involvement of other phosphorylation sites in PTEN or TNS3–ABD binding to DLC1 and PI3K, we employed recombinant glutathione *S*-transferase (GST)–PTEN, GST–TNS3–ABD or a mutant to pull down FLAG-DLC1 or FLAG-PI3K–p85 from HEK293 cells. The PTEN mutants T319E and T319Y, but not the wt protein, pulled down DLC1 effectively, indicating that phosphorylation of Thr319 or its swap with a Tyr in the ABD enabled PTEN binding to DLC1. Interestingly, the substitution of Tyr321 by Glu (to mimic PTEN-Thr319 phosphorylation) promoted TNS3–ABD binding to DLC1 while replacing Thr323 with either Ala or Glu abolished binding (Fig. 3e). In contrast, only the specificity-switching Y321T and the pThr-mimicking T323E mutants of ABD were capable of binding PI3K. Intriguingly, the PTEN mutant T319E failed to bind PI3K–p85, suggesting that phosphorylation of Thr319 inhibits the PTEN–PI3K interaction (Fig. 3f). Taken together, we have identified a ‘phosphorylation switch' (or P-switch) in PTEN and TNS3 that dictates their binding preference for DLC1 or PI3K (Fig. 3g). The P-switch is enabled by phosphorylation of TNS3 at Thr323 and PTEN at Thr319 and Thr321 in their respective C2 domains.

### Regulation of the global activity of RhoA and Rac1

**Figure 4:**
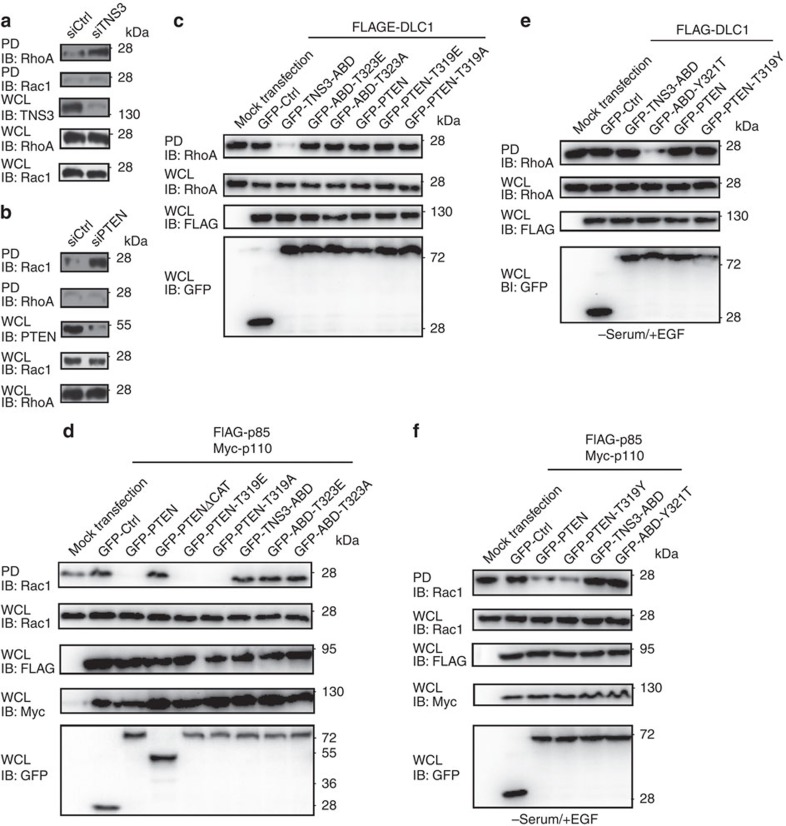
Effects of the P-switch on the global activities of RhoA and Rac1. (**a**) Depletion of TNS3 from MCF10A activated RhoA, but not Rac1. GTP-bound RhoA or Rac1 was pulled down (PD) with Rhotekin-RBD or PAK-PBD agarose beads from cell lysates and blotted with an anti-RhoA or anti-Rac1 antibody. WCL IB was included to show the total levels of TNS3, RhoA and Rac1. (**b**) Depletion of PTEN augmented the activity of Rac1, but not RhoA. The same set of experiments as in **a** were carried out in MCF10A cells transfected with a PTEN-specific siRNA or a scrambled oligo (siCtrl). (**c**) Overexpression of TNS3–ABD, but not PTEN nor a mutant of either protein, led to inactivation of RhoA. HEK293 cells expressing GFP fusion TNS3–ABD, PTEN or the indicated mutants were subjected to a Rhotekin-PD assay. (**d**) PTEN and its point mutants, but neither a PTEN truncation mutant missing the catalytic domain (▵CAT) nor TNS3–ABD and its point mutants, repressed Rac1 activity. A PAK-PBD PD assay was used to assess Rac1 activity in HEK293 cells co-expressing PI3K (that is, FLAG-p85 plus Myc-p110) and the indicated PTEN or TNS3–ABD proteins. (**e**) The specificity-switching mutant, TNS3–ABD–Y321T, but neither PTEN, PTEN–T319Y, nor TNS3–ABD, inactivated RhoA. DLC1-expressing HEK293 cells were serum starved and treated with EGF for 30 min before the Rhotekin-PD assay. (**f**) wt PTEN and the specificity-switching mutant, PTEN–T319Y, but neither TNS3–ABD nor the ABD–Y321T mutant, inhibited the gross activation of Rac1. Cells were treated the same way as in **e**. Data shown are representative of three independent experiments.

To understand how the dynamic protein–protein interactions enabled by the P-switch regulate cell migration, TNS3 or PTEN was knocked down by short interfering RNA (siRNA) from MCF10A cells. Depletion of TNS3 led to a marked activation of RhoA, but having no effect on Rac1 (Fig. 4a). In contrast, depletion of PTEN resulted in the activation of Rac1 without affecting RhoA (Fig. 4b). Co-expression of DLC1 and the TNS3–ABD mutants T323A or T323E failed to inactivate RhoA in the same way as the wt ABD, due likely to inability of the mutants to bind DLC1. Remarkably, neither the PTEN mutant T319E, which bound DLC1 constitutively (Fig. 3a), nor the binding-defective mutant T319A, had any effect on RhoA when co-expressed with DLC1 (Fig. 4c). This suggests that the function of the PTEN–DLC1 interaction, occurring only when PTEN is phosphorylated at Thr319, is likely to sequester PTEN. Moreover, the inhibitory effect of PTEN on Rac1, which is dependent on the catalytic domain (Fig. 4d), is not coupled to PI3K binding. Intriguingly, neither TNS3–ABD nor a Thr323 mutant was able to inactivate Rac1 in HEK293 cells cultured in normal growth medium (Fig. 4d).

In serum-starved cells with EGF stimulation, however, the activity of RhoA was no longer affected by TNS3–ABD (Fig. 4e). Apparently, this was due to the phosphorylation and subsequent dissociation of TNS3–ABD from DLC1 (Fig. 1d). Remarkably, the specificity-switching mutant TNS3–ABD–Y321T, which bound DLC1 in the presence of EGF (Fig. 3a), abolished RhoA activation. In contrast, neither the wt PTEN nor the PTEN–T319Y mutant had any effect on RhoA (Fig. 4e), reconfirming the earlier observation that PTEN is not directly involved in regulating RhoA activity (Fig. 4b). Intriguingly, while Rac1 was activated in PI3K-overexpressing HEK293 cells following EGF stimulation, it was inactivated by the co-expression of either the wt PTEN or the T319Y specificity-switching mutant. Nevertheless, neither wt TNS3–ABD nor the specificity-switching mutant Y321T was able to inactivate Rac1 (Fig. 4f). These data reinforce the notion that the global activity of RhoA is controlled by DLC1 via TNS3, independently of PTEN; and the gross activity of Rac1 is controlled by PTEN, irrespective of TNS3.

### The P-switch controls spatial activation of Rac1 and RhoA

**Figure 5:**
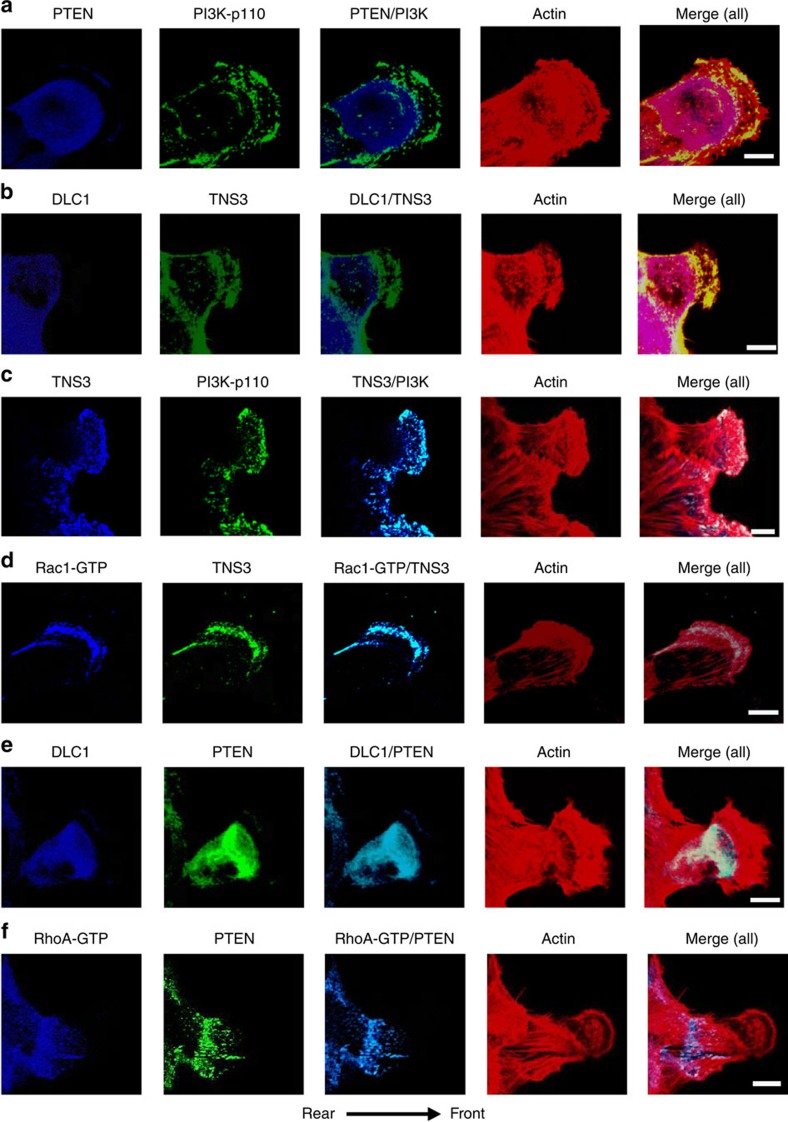
The P-switch controls spatiotemporal activation of RhoA and Rac1. (**a**) Front–rear-polarized localization of PI3K and PTEN. EGF-treated MCF10A cells were co-immunostained for PTEN (blue) and PI3K-p110 (green). Actin was stained with rhodamine-phallodin (red). (**b**) Front–rear-polarized localization of TNS3 and DLC1 in cells under the same condition as in **a**. (**c**) TNS3 and PI3K-p110 co-localized to the leading edge of migrating cells. Cells as in **a** were immunostained for TNS3 (blue) and PI3K-p110 (green). (**d**) Rac1-GTP and TNS3 co-localized to the lamellipodial protrusion at the leading edge of a migrating cell. Cells were co-stained for Rac1-GTP (blue), TNS3 (green) and actin (red). (**e**) DLC1 (blue) and PTEN (green) co-localized to the posterior of a migrating cell. (**f**) RhoA-GTP (blue) and PTEN (green) co-localized to the posterior of a migrating cell. Images shown are representative of three independent experiments. Arrow indicates direction of migration. Scale bar, 10 μm.

The apparent decoupling of binding from global RhoA and Rac1 activation implies that the PTEN–DLC1 and TNS3–PI3K complexes, formed in response to EGF signalling, may provide a mechanism for spatiotemporal activation of RhoA and Rac1 conducive to directional migration. We tested this idea by first examining whether the components of the switch were mobilized in response to EGF treatment. Confocal immunofluorescence (IF) microscopy showed that, while PI3K and PTEN were evenly distributed and co-localized in MCF10A cells in the absence of EGF stimulation (Supplementary Fig. 10a), the former, but not the latter, was found in the anterior of migrating cells(Fig. 5a). Similarly, whereas TNS3 and DLC1 co-localized with each other and with paxillin in resting cells (Supplementary Fig. 10b,c), TNS3, but not DLC1, moved to the membrane protrusions in EGF-stimulated cells (Fig. 5b). The distinct, dynamic movements of PTEN and TNS3 in response to EGF signalling were captured by confocal time-lapse images showing that TNS3 and PI3K (p85) gradually formed a crescent at the membrane protrusions of migrating cells, whereas PTEN and DLC1 were found primarily in the cell body (Supplementary Fig. 11). Moreover, TNS3 was found co-localized with PI3K (Fig. 5c) and Rac1-GTP (Fig. 5d) in lamellipodial protrusions at the leading edge, whereas PTEN immunofluorescence overlapped with that of DLC1 (Fig. 5e) and RhoA-GTP (Fig. 5f) in the posterior or at the trailing edge of the migrating cell. These data, not observed in cells without EGF stimulation or when Mek1/2 was inhibited (Supplementary Fig. 10d–g), imply that TNS3, on phosphorylation, relocates to the anterior to form a complex with PI3K for Rac1 activation at the membrane protrusions. Conversely, phosphorylated PTEN moves to the posterior of the cell to form a complex with DLC1 for localized RhoA activation. Therefore, EGF signalling led to not only an exchange of binding partners for TNS3 and PTEN, but also polarized distribution of the TNS3–PI3K and PTEN–DLC1 complexes and spatial activation of Rac1 and RhoA.

### The P-switch is necessary for EGF-induced migration

**Figure 6:**
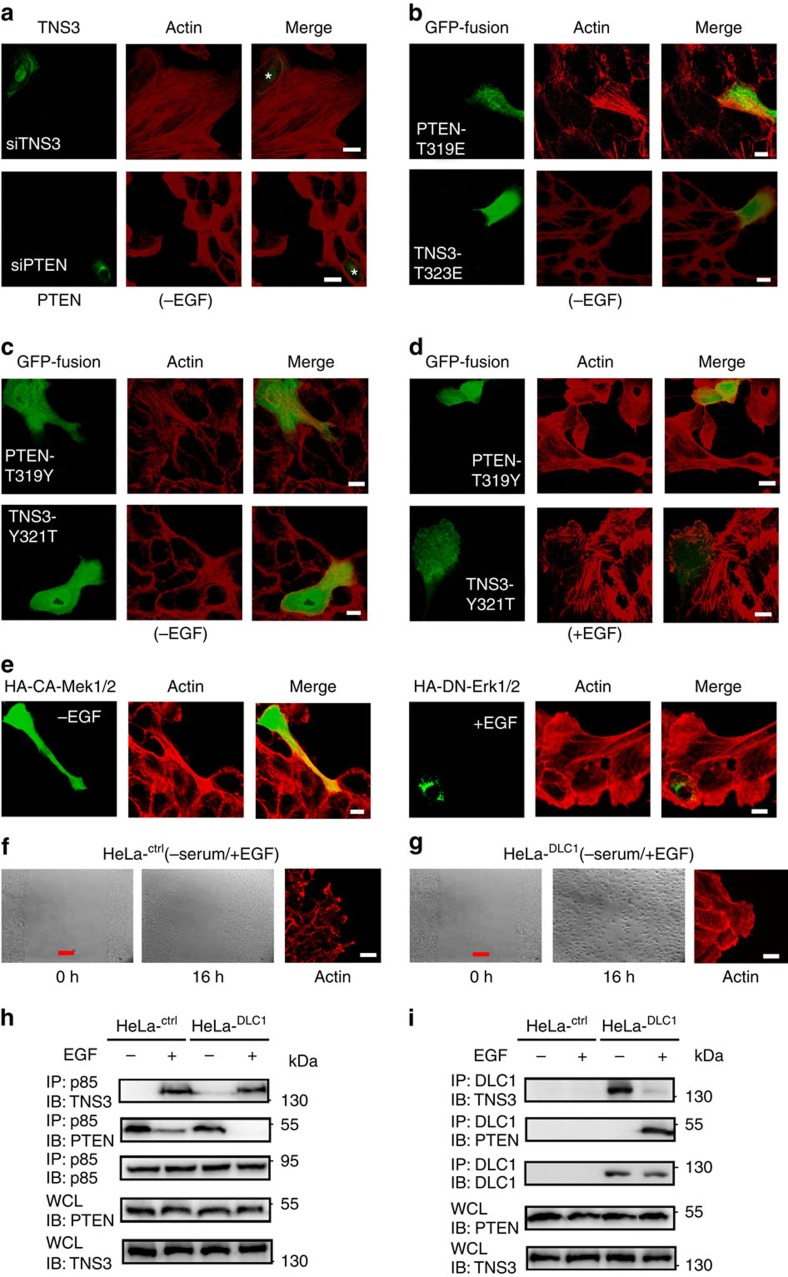
Integrity of the P-switch is critical for EGF-induced cell migration. (**a**) Depletion of TNS3 markedly enhanced actin stress fibre formation, whereas the knockdown of PTEN dramatically promoted lamellipodial formation. MCF10A cells were transfected with siRNA specific for TNS3 or PTEN and serum starved before immunostaining and confocal imaging: TNS3 and PTEN, green; actin, red. Each microscopic field contains one non-transfected cell identified by positive IF for TNS3 or PTEN (marked by asterisks). (**b**–**e**) Abnormal cell morphology engendered by the expression of a PTEN, TNS3, Mek1/2 or Erk1/2mutant. MCF10A cells were transfected with plasmids encoding the indicated proteins, serum starved and imaged after 30 min incubation with EGF (+EGF) or buffer (−EGF). Note that the green fluorescence in **b**–**e** indicates transfected cells. (**f**,**g**) Phase-contrast images of HeLa or HeLa^DLC1^ cells at 0 and 16 h after wounding by scratch. Right, confocal images of the same cells with actin staining (red). Cells were cultured in serum-free medium with EGF. Images shown in **a**–**g** are representative of four independent experiments. Scale bar, 10 μm (white), 100 μm (red). (**h**,**i**) The defective P-switch in HeLa was restored in the HeLa^DLC1^ cells that stably express DLC1. Both the HeLa and HeLa^DLC1^ cells, treated (+) or not (−) with EGF, were subjected to IP/IB to evaluate the dynamic binding among TNS3, PTEN, PI3K and DLC1.

To determine the role of the P-switch in cell migration, we investigated if disrupting the dynamic interaction network of DLC1, TNS3, PI3K and PTEN would lead to aberrant changes in the actin cytoskeleton and cell migration. Depletion of TNS3 from MCF10A cells induced the formation of actin stress fibres without EGF stimulation, apparently through RhoA activation; depletion of PTEN, on the other hand, augmented lamellipodia, likely via increased Rac1 activity (Fig. 6a). In contrast, actin stress fibres or lamellipodia were not observed in control cells with normal levels of TNS3 or PTEN (Fig. 6a; marked with asterisks) or in cells transfected with a scrambled siRNA (Supplementary Fig. 10h). Expression of the constitutively binding PTEN mutant T319E induced the formation of actin stress fibres, possibly by displacing endogenous TNS3 from DLC1 (Fig. 6b). Expression of TNS3–T323E, on the other hand, resulted in increased lamellipodium formation, likely by displacing endogenous PTEN from PI3K (Fig. 6b). The specificity-switching PTEN–T319Y mutant promoted the formation of actin stress fibres in the absence of EGF, while the TNS3–Y321T mutant enhanced lamellipodium formation (Fig. 6c). In the presence of EGF, however, the PTEN–T319Y mutant inhibited the formation of lamellipodia, presumably by displacing endogenous TNS3 from PI3K; the TNS3–Y321T mutant blocked the formation of actin stress fibres, likely by competing against the PTEN–DLC1 interaction (Fig. 6d). By comparison, the GFP-vector control, the wt PTEN and TNS3, and the binding-defective mutants PTEN–T319A and TNS3–T323A, effected no significant change on cell morphology (Supplementary Fig. 12). Intriguingly, expression of the constitutively active Mek1/2 in MCF10A cells led to enhanced stress fibre formation and enabled cell stretching; expression of dominant negative (DN) Erk1/2, on the contrary, inhibited stress fibre formation and resulted in the round cell morphology (Fig. 6e).

To determine whether the integrity of the P-switch is necessary for EGF-driven cell migration, we took advantage of several epithelial cancer cell lines with differential expression of the four proteins comprising the switch. While MDA-MB-231 contains all four components of the switch at levels comparable to those found in MCF10A, MCF7 expresses less TNS3, and the MDA-MB-468, HeLa and A549 cells are devoid of DLC1 and/or PTEN (Supplementary Fig. 13a). Under EGF stimulation, MDA-MB-231 cells formed large, unipolar lamellipodial protrusions similar to those observed in MCF10A cells, whereas the remaining cell lines featured multiple, randomly projecting actin bundles (Supplementary Fig. 13b). Consequently, MCF10A and MDA-MB-231 were capable of healing scratch wounds in response to EGF stimulation, while the remaining types of cells failed to or were much less efficient in wound repair under the same condition (Supplementary Fig. 13c). This suggests that the P-switch is necessary for epithelial cell migration driven by EGF. Interestingly, serum induced the migration of the cancer cells, but not MCF10A (Supplementary Fig. 13c), suggesting that the former have acquired the ability to migrate in response to factors other than EGF that are present in the serum.

We next tested whether reconstitution of the switch in HeLa cells by forced expression of DLC1 would enable directional migration. Remarkably, the restoration of DLC1 resensitized HeLa cells to EGF-stimulated migration as evident in the ability of the HeLa^DLC1^ cells to repair scratch wounds and form large, unipolar lamellipodia (Fig. 6f,g) in response to EGF. The inability of the parent HeLa cells to respond to EGF could be due to an incomplete P-switch as a significant amount of PTEN was still bound to PI3K–p85 even after EGF stimulation for 30' (Fig. 6h). The switch was, however, complete with the restoration of DLC1 expression (Fig. 6h,i).

### The P-switch controls directional migration induced by PDGF

**Figure 7:**
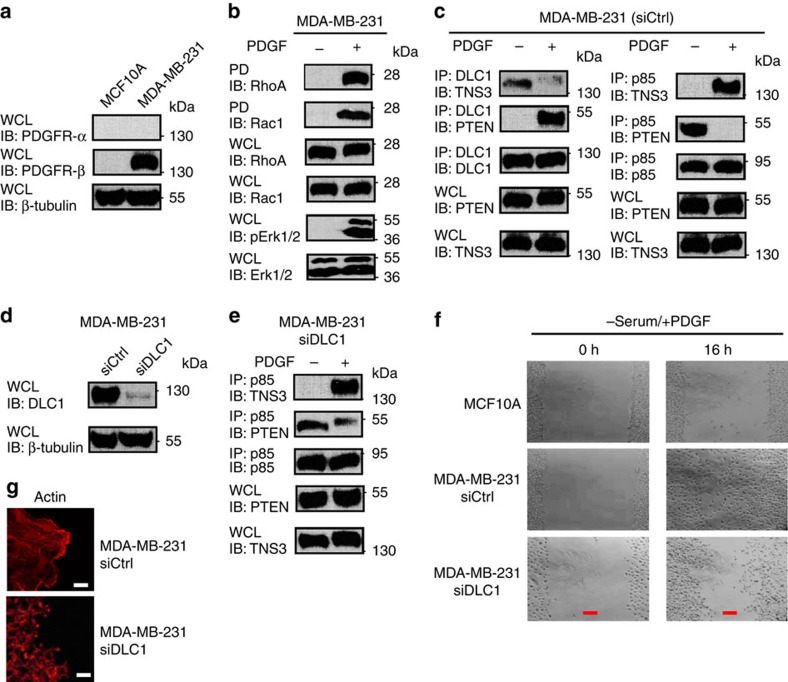
PDGF regulates directional cell migration via the P-switch. (**a**) A western blot showing the expression of PDGFR-β in MDA-MB-231, but not in MCF10A. (**b**) Acute PDGF treatment (30 min) following serum starvation led to phosphorylation of Erk1/2 and activation of RhoA and Rac1. (**c**) PDGF regulates the dynamic interactions of DLC1, TNS3, PTEN and PI3K. (**d**) A western blot showing the knockdown of DLC1 in MDA-MB-231 cells. (**e**) A ‘defective' P-switch in DLC1-depleted MDA-MB-231: PI3K–p85 was found to bind both PTEN and TNS3 in the absence of DLC1 following PDGF stimulation. (**f**) Phase-contrast images of MCF10A and MDA-MB-231 cells transfected with control or DLC1-specific siRNA at 0 and 16 h after wounding by scratch. Cells were cultured in serum-free medium with PDGF. (**g**) Confocal images of MDA-MB-231 cells with or without depletion of DLC1 showing the distinct patterns of actin network at the edge of the scratch wound. Scale bar, 10 μm (white), 100 μm (red). Data shown are representative of three independent experiments.

The P-switch may regulate directional migration under different motility stimuli that signal through the MAPK pathway. We explored this possibility by examining PDGF-induced migration of the MDA-MB-231 cells that express a high level of PDGFR-β^45^ (Fig. 7a). PDGF stimulation following serum starvation led to a marked increase in both RhoA and Rac1 activation (Fig. 7b). Moreover, PDGF treatment led to a switch of binding partners for DLC1 from TNS3 to PTEN, and for PI3K–p85 from PTEN to TNS3 (Fig. 7c). In contrast to the control MDA-MB-231 cells, depletion of DLC1 by siRNA led to an incomplete switch of binding partners for PI3K–p85 (Fig. 7d,e), failure in wound repair (Fig. 7f) and the formation of smaller, disorganized actin projections (Fig. 7g).

## Discussion

**Figure 8:**
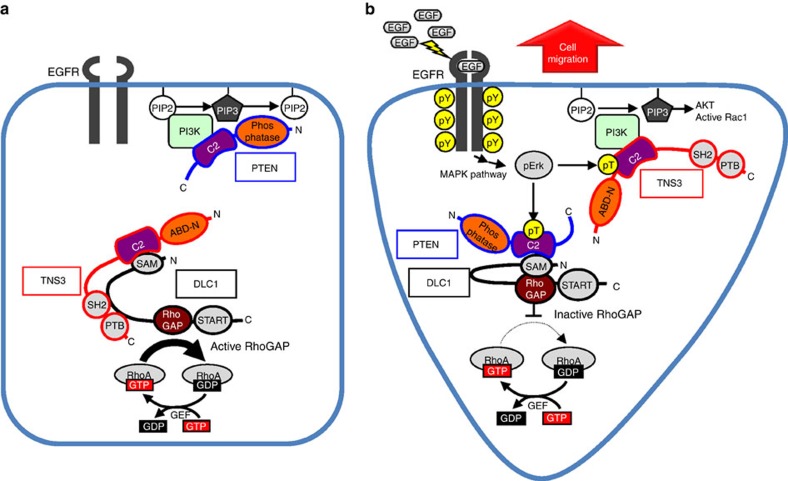
A schematic model of the P-switch in directed cell migration. The model depicts how a molecular switch comprising TNS3, DLC1, PI3K and PTEN controls the directional migration of epithelial cells via spatiotemporal activation of Rac1 and RhoA. **a**, without EGF stimulation; **b**, with EGF.

Our work identifies a novel mechanism by which the EGF/PDGF signalling drives the directional migration of epithelial cells (Fig. 8). In resting cells, the global cellular activities of both RhoA and Rac1 are kept at a low level by the DLC1–TNS3 and PI3K–PTEN complexes, respectively. Specifically, binding by TNS3 (via its C2 domain) to DLC1 releases an autoinhibitory interaction between the SAM and RhoGAP domains of the latter, leading to GAP activation and RhoA inactivation^18^. On the other hand, Rac1 activity is repressed by reduced PI3K signalling as a result of the PI3K–PTEN interaction. Following EGF or PDGF stimulation, however, both the global and local activities of Rac1 and RhoA are increased. Binding of the EGF to its receptor (EGFR) activates the MAPK signalling pathway that culminates in the activation of Mek1/2 and Erk1/2 (ref. 46). This leads to Thr phosphorylation of TNS3 and PTEN on their respective C2 domains, which, in effect, reverses their binding-partner specificity. Phosphorylation of TNS3 on Thr323 changes its binding preference from DLC1 to PI3K. Although binding of phosphorylated TNS3 to PI3K does not affect the activity of the latter, it displaces PTEN, a negative regulator of PI3K, from the membrane protrusion and thereby, resulting in increased local PIP3 concentration and Rac1 activity. On the other hand, PTEN, when phosphorylated at the Thr319 and Thr321 sites, dissociates from PI3K and is recruited instead by DLC1. Unlike the TNS3–DLC1 complex, the phospho-PTEN–DLC1 interaction is incapable of activating the RhoGAP function of DLC1; rather, it serves to keep DLC1 in an auto-inhibited state to facilitate local RhoA activation^18^,^47^. Reciprocally, DLC1, which is found mainly at focal adhesions in the lateral or posterior region of a migrating cell, sequesters phosphorylated PTEN away from the leading edge. The switch of binding partners is concomitant to translocation of the TNS3–PI3K complex to the anterior and the PTEN–DLC1 complex to the posterior of the cell to promote Rac1 and RhoA activation in a spatiotemporal fashion to initiate directional migration.

It is remarkable that a single residue, namely Thr321 in the PTEN and Tyr319 in the TNS3, dictates their respective binding specificity. Swapping these residues completely reversed binding-partner preference for PTEN and TNS3. The C2 domain is a large family of lipid-binding module^48^,^49^; nevertheless, the PKCδ C2 domain has been shown to bind the glycoprotein CDCP1 in a phosphotyrosine-dependent manner^50^. Our work suggests that some C2 domains are bona fide protein–interaction modules as shown here for the PTEN and TNS3 C2 domains.

While acute stimulation of MCF10A triggers the P-switch, prolonged (>1 h) stimulation induces a ‘transcriptional switch' (or T-switch) that involves a decrease in TNS3 expression and a simultaneous increase in CTEN expression^18^,^35^. How, then, would the P-switch be affected by the T-switch? We addressed this question by examining the dynamic interactions of DLC1, PI3K, PTEN, TNS3 and CTEN at different time points during 16 h of continuous EGF treatment of MCF10A cells. While there was a clear exchange of binding partners for DLC1 and PI3K at 30-min EGF stimulation, the situation appeared to be reversed at 1 h. Curiously, DLC1 was found to rebind TNS3 and PTEN reassociate with PI3K–p85 at 1 h of EGF stimulation (Supplementary Fig. 14). This indicates that the P-switch is turned on and then off during the first hour of EGF stimulation, due likely to the dynamic nature of phosphorylation. At 16 h, the P-switch is no longer intact as TNS3 is replaced by CTEN. At this point, we found DLC1 associated with both PTEN and CTEN, whereas PI3K decoupled from PTEN. While the detailed mechanism of interplay between these two switches remains to be defined, both switches play an essential role in directional migration of epithelial cells.

Unlike chemotaxis, EGF or PDGF, when applied uniformly to the cell culture, does not provide a directional cue. Therefore, directional migration triggered by EGF/PDGF must involve intrinsic cellular directionality^1^. We showed that EGF signalling contributed to the formation of front–rear polarity in MCF10A cells. The formation of the front–rear-polarized localization pattern for PI3K and PTEN may play a critical role in initiating directional migration. Rac1 activation at the membrane protrusion may be regulated by PIP3 (ref. 51). In support of this notion, Tiam1, Vav2 and Asef, Rac GEFs that contain a PIP3-binding PH domain, have been shown to activate Rac1 in response to EGF^52^,^53^. Nevertheless, alternative pathways may also regulate Rac1 activation^54^.

How is RhoA activation regulated in a spatially restricted manner? We showed that the activation of RhoA in the body or rear of migrating cells was controlled by the DLC1–PTEN complex, which serves multiple roles. First, DLC1 is normally kept inactive by an auto-inhibition mechanism^18^,^47^. Because PTEN was incapable of activating DLC1, the PTEN–DLC1 complex would be expected to promote local activation of RhoA, likely at sites of focal adhesion where DLC1 is normally found^15^,^18^,^44^. Second, PTEN, even when phosphorylated, could still inhibit Rac1 activation. This is expected given that the inhibitory function of PTEN on Rac1 was depended on the catalytic, rather than the C2 domain of the former. Thus, the PTEN–DLC1 complex would decrease Rac1 activity in the centre or rear of a migrating cell. And third, the antagonism between Rac1 and RhoA^55^,^56^,^57^ may provide an additional layer of control over the P-switch to ensure the spatial activation of Rac1 and RhoA during directional cell migration. In this regard, active RhoA in the posterior of a migrating cell would inhibit Rac1 activation in the same region through the Rho-kinase ROCK^58^; conversely, robust Rac1 activation would counter the activation of RhoA at the leading edge^59^,^60^,^61^.

While it remains to be determined whether the P-switch is a general mechanism underlying motility stimuli that activate the MAPK pathway, we showed that both EGF- and PDGF-induced epithelial cell migration depends on the switch. Moreover, we demonstrated that the integrity of the P-switch is essential for directional cell migration. HeLa cells, which feature an incomplete P-switch due to the lack of DLC1 expression, were unable to initiate directional migration in response to EGF. Interestingly, the restoration of DLC1 expression in HeLa, which fully reconstituted the P-switch, enabled EGF-dependent migration. In a completely opposite fashion, depletion of DLC1 from MDA-MB-231 cells rendered them irresponsive to PDGF-induced directional migration.

Although it is possible that the P-switch, or a variation of it, may play a role in cell migration downstream of growth factors other than EGF and PDGF, cell migration would necessarily involve additional regulatory pathways depending on cellular and environmental contexts. In this regard, it is worth noting that Lowy and colleagues^62^ have shown recently that DLC1 may be activated by CDK5-mediated phosphorylation of a linker sequence between the SAM and RhoGAP domains, distinct from the mechanism of activation by TNS3 (ref. 18). Moreover, directional sensing by neutrophils during chemotaxis is dependent on SHIP1, rather than PTEN^63^. The issue is further compounded by the fact that cells in their physiological setting may encounter multiple motility stimuli at any given time. This may account, in part, for the differences in cell motility of cells in serum-containing and serum-free media (Supplementary Fig. 13c). An important question to address in the future is how these diverse signals are integrated to coordinate cell migration.

The P-switch identified herein may have important implications to animal development and wound healing for which directional cell migration plays a critical role^51^,^61^. It is also intriguing that the proteins comprising the switch are either oncogenic or tumour suppressive. It is possible that the deregulation of the P-switch plays a part in cancer metastasis. In this regard, it is interesting to note the breast cancer lines with different metastatic potentials such as MDA-MB-231 and MDA-MB-468 (ref. 64) behaved very differently in wound-healing assays (Supplementary Fig. 13c). Apparently, further studies are required to delineate the role of the P-switch in cancer metastasis or other pathological conditions.

## Methods

### DNA constructs and mutagenesis

Human DLC1 and TNS3 were subcloned into the pcDNA3.1 and pEGFPC3 vectors. TNS3-C2 and TNS3–ABD-N were generated by PCR cloning from pEGFPC3-TNS3 and into the same vector via the BglII and SalI sites. The pEGFPC1-PTEN and pEGFPC1-PTENΔCAT constructs were kindly provided by Monilola A. Olayioye (University of Stuttgart, Stuttgart, Germany). Plasmids encoding the truncated fragments of PTEN, pEGFPC1-PTEN-CAT, pEGFPC1-PTEN-C2, pEGFPC1-PTEN-N (1–350 a.a.) and pEGFPC1-PTEN-C (351–403 a.a.) were generated by PCR cloning from the pEGFPC1-PTEN construct via BamHI site. PTEN and TNS3–ABD were amplified by PCR and subcloned into the pGEX6P3 GST expression vector (GE Healthcare) via BamHI or SalI site. The pEGFPC1-DLC1 construct was provided by Michael Mowat (Manitoba Institute of Cell Biology, Winnipeg, Canada). The pEGFPC1-p85, pFLAG3-p85α and pMyc-p110α constructs were a generous gift from Deborah Anderson (Saskatchewan Cancer Agency, Saskatoon, Canada). The pCEP4-HA-CA-Mek1/2 and pCEP4-HA-DN-Erk1/2constructs were kindly provided by Jong-In Park (Medical College of Wisconsin, Milwaukee, WI, USA).

Site-specific mutants of PTEN, PTEN-C2, TNS3, TNS3–ABD and TNS3-C2 were created using the QuikChange Site-Directed Mutagenesis Kit (Stratagene) according to the manufacturer's instructions using the primers listed in Supplementary Tables 1 and 2. All constructs and mutations were verified by DNA sequencing.

### Reagents and antibodies

Unless indicated otherwise, all chemical reagents were purchased from Sigma-Aldrich. Glutathione Sepharose 4 Fast Flow was purchased from GE Healthcare. All restriction enzymes were purchased from Fermentas Life Sciences. Mouse anti-human PDGFR-α (MAB322, R.D. Systems) and Mouse anti-human PDGFR-β (MAB1263, R.D. Systems) were gifts from Zia Khan (Western University, Canada). The following antibodies were purchased from Santa Cruz Biotech Inc.: rabbit anti-paxillin (H-114, SC-5574), rabbit anti-β-tubulin (H-235; sc-9104), rabbit anti-DLC1 (H-260; sc-32931), mouse anti-DLC1 (C-12; sc-271915), rabbit anti-TNS3 (tensin-3; sc-134908), goat anti-TNS3 (tensin-3; T-18; sc-55155), rabbit anti-PTEN ((C-20)-R; sc-6817-R), mouse anti-PTEN (A2B1; sc-7974), rabbit anti-Myc (A-14; sc-789), mouse anti-RhoA (26C4; sc-418), mouse anti-HA (F-7; sc-7392), rabbit anti-GST(Z-5; sc-459) and mouse anti-GFP (B-2; sc-9996). Rabbit anti-p44/42 MAPK (Erk1/2; cat # 9102), mouse anti-Phospho-p44/42 MAPK (Thr202/Tyr204; cat # 9106), mouse anti-Phospho-Tyrosine(cat # 9411S) and mouse anti-Phospho-Threonine (cat # 9386)antibodies were purchased from Cell Signaling Technology Inc. Rabbit anti-PI3K p110α (cat # 04-399), mouse anti-PI3K p85α (cat # 05-212) and mouse anti-Phosphoserine (cat # 05-1000) antibodies were obtained from Millipore. A mouse anti-Rac1 antibody (cat #ARC03) was purchased from Cytoskeleton. Rabbit anti-GFP (cat # G1544) and mouse anti-FLAG (M2; cat # F1804) antibodies were obtained from Sigma-Aldrich. Mouse anti-active RhoA-GTP (cat # 26904) and anti-active Rac-GTP (cat # 26903) antibodies were from NewEast Biosciences. Goat anti-mouse IgG (H+L)-HRP (cat # 170-6516) and anti-rabbit IgG (H+L)-HRP (cat # 170-6515) conjugates were obtained from Bio-Rad Laboratories Inc.

### Cell culture

HEK293, A549, HeLa, MDA-MB-231, MCF7 and MCF10A cells were obtained from American Type Culture Collection (ATCC, Manassas, VA). MDA-MB-468 was a kind gift from Alison Alan (London Regional Cancer Program, London, ON, CA). HEK293, A549, HeLa and MCF7 cells were maintained in Dulbecco's Modification of Eagle's medium (DMEM) containing antibiotics and 10% fetal bovine serum (FBS; Sigma-Aldrich). MDA-MB-231 cells were maintained in DMEM: F12 containing antibiotics and 10% FBS. MDA-MB-468 cells were maintained in α-MEM containing antibiotics and 10% FBS. MCF10A cell full culture medium contained DMEM: F12 medium supplemented with antibiotics, EGF (10 ng ml^−1^), insulin (10 μg ml^−1^), cholera toxin (1 μg ml^−1^), hydrocortisone (1 μg ml^−1^) and heat-inactivated horse serum (5%; Invitrogen).

Serum-free (starved) medium contained no serum and growth factors. EGF treatment medium contained 20 ng ml^−1^ EGF in the serum-free medium. For EGF treatment, cells were incubated with EGF (20 ng ml^−1^) treatment medium for 5, 15, 30 min or 16 h after 16 h serum starvation at 37 °C in 5% CO_2_. PDGF treatment medium contained 30 ng ml^−1^ PDGF-BB (Sigma-Aldrich) in the serum-free medium. For PDGF treatment, the cells were incubated with PDGF treatment medium for 30 min or 16 h after 16 h serum starvation at 37 °C in 5% CO_2_. For U0126 (Cell Signaling Technology Inc.) treatment, cells were incubated with 10 μM U0126 for 45 min at 37 °C in 5% CO_2_. For Src Inhibitor 1 (Sigma-Aldrich) treatment, cells were treated with 50 nM Src-I1for 45 min at 37 °C in 5% CO_2_.

Cell transfection medium contained no antibiotics. Transient transfections were carried out with jet-PEI (PolyPlus-Transfection; Illkirch, France) according to the manufacturer's instructions.

### IP and western blot

Cells were lysed in cold lysis buffer (1% NP-40, 50 mM Tris pH 7.4, 150 mM NaCl, 2 mM EDTA, 50 mM NaF, 10% Glycerol and the Halt Protease and Phosphatase Inhibitor Cocktail (Thermo Scientific) diluted at 1:100. To prepare MCF10A cell lysates, cell pellets were sonicated in 0.2-ml lysis buffer on ice, and the lysate was spun down at 16,000*g* for 15 min at 4 °C. The supernatant was collected and the protein concentration was determined using the Bio-Rad Protein Assay Kit. After clearing the lysate with an appropriate pre-immune serum and protein G (Roche), IP was performed using indicated antibodies (apply 3 μg antibody per reaction). The immunoprecipitates were resolved on SDS–polyacrylamide gel electrophoresis. On blotting the gel to polyvinylidene difluoride membrane, the proteins were detected by IB with appropriate secondary antibodies and visualized by ECL. The dilution of the first antibodies is 1:1,000, and that of the HRP-conjugated secondary antibodies is 1: 5,000. Uncropped western blot images are provided in Supplementary Fig. 15.

### Bacterial protein expression and GST pull down

The BL21 strain of *E. coli* was transformed with pGEX6P3-PTEN-WT or pGEX6P3–TNS3–ABD-WT or a mutant. Protein expression was induced with 0.1 mM isopropyl-β-D-thiogalactoside for 16 h at 18 °C when OD600 reached 0.6. The bacterial culture was harvested and pellets were resuspended in PBS containing complete protease inhibitors (Roche). The suspension was sonicated for three times (10 s each) on ice. Triton-X-100 was added to a final concentration of 1% and the lysates were centrifuged at 16,000*g* for 15 min under 4 °C. Purification of GST-tagged proteins was performed with glutathione agarose (GE Healthcare). The resin was washed in PBS for three times. Pull down was performed by incubating GST-tagged proteins with lysate of HEK293 expressing FLAG-DLC1 or FLAG-p85 for 3 h, followed by washing the beads with lysis buffer for three times at 4 °C. Proteins bound to GST-beads were resolved on SDS–polyacrylamide gel electrophoresis and identified by western blot as described above.

### Knockdown of protein expression by small interference RNA

SiRNAoligos targeting *pten* (sc-29459), *dlc1*(sc-43725) and the control siRNA (sc-44236) were obtained from Santa Cruz Biotech Inc. The accession number of On-Target Plus SMART pool siRNA oligonucleotides against *tensin-3* (Thermo Fisher Scientific-Dharmacon) was L-009997-00. SiRNA oligos were transfected into subconfluent MCF10A or MDA-MB-231 cells using the DharmaFECT 1 Transfection Reagent (T-2001-01) according to the manufacturer's instructions.

### RhoA or Rac1 activation assay

RhoA or Rac1 activities were measured using the Rho or Rac Activation Assay Biochem Kit (Cytoskeleton). Cells at 40–60% confluency were treated with EGF (20 ng ml^−1^) or transfected with the indicated siRNA or plasmids. After culturing for 30 min (EGF treatment) or 24 h (transient transfection), the cells were washed in ice-cold PBS and lysed. Equal amounts of whole-cell lysates were incubated with 20 μg Rhotekin-RBD (Rho Binding Domain of Rhotekin) or PAK-PBD (p21 Binding Domain of p21 Activated Kinase 1) beads for 1 h at 4 °C. The beads were washed three times with washing buffer, and the bound RhoA or Rac1 proteins were analysed by western blots using an anti-RhoA or anti-Rac1 antibody.

### Generation of the HeLa^DLC1^ stable cell line

HeLa cells were transfected with pcDNA3.1(+) or pcDNA3.1(+)-DLC1 by the jet-PEI (PolyPlus-Transfection; Illkirch, France). The transfected cells were cultured in medium containing G418 (400 μg ml^−1^) starting 24 h after transfection. Following western blotting to measure DLC1 expression, the colonies of interest were cultured in medium containing 400 μg ml^−1^ G418 for ∼2 months. The resulting line that stably express DLC1, designated HeLa^DLC1^, were maintained in culture medium containing 200 μg ml^−1^ G418.

### Wound-healing assay

Cells at 100% confluency post 16 h serum starvation were scratched using a 200 μl pipette tip and the cell debris were washed away with PBS. Cells were then incubated for 16 h with or without serum or EGF or PDGF-BB. Images were captured at the beginning (0 h) and at regular intervals (16 h) using the Infinity Capture Imaging System (Lumenera Corporation) on the Motic AE31 Inverted Microscope (Matic Microscope).

### Confocal immunofluorescence microscopy

Cells grown in 35-mm glass bottom dishes (P35G-1.0-14-C, MatTek) were treated with EGF (20 ng ml^−1^) for 30 min after 16 h serum starvation or transfected with appropriate constructs or siRNA oligos and incubated for 24 h. Cells were then fixed in 2% formaldehyde at room temperature. After rinsing in PBS, cells were incubated with anti-Paxillin (rabbit at 1:100 dilution), anti-DLC1 (mouse at 1:100 dilution), anti-PTEN (rabbit at 1:100 or mouse at 1:200), anti-TNS3 (tensin-3; rabbit at 1:100 or goat at 1:50), anti-PI3K-p110 (rabbit at 1:200), anti-HA (mouse at 1:200), anti-active Rac1-GTP (mouse at 1:200) or anti-active RhoA-GTP (mouse at 1:200) antibodies for 3 h at room temperature. Samples were then incubated with the corresponding Alexa Fluor-488, -546 or -633-conjugated secondary antibodies (Invitrogen-Molecular Probes, 1:1,000) for 1 h followed by incubation with rhodamine phalloidin (Invitrogen, 1:50) or 4′,6-diamidino-2-phenylindole (Calbiochem, 1:1,000) for 30 min. Samples were visualized on a ZEISS LSM 510 META/Confocor2 microscope(Carl Zeiss MicroImaging) with pinhole set at 1 airy unit using 488, 546 and/or 633 nm excitation and a × 63/1.4 oil objective lens.

### Time-lapse microscopy

MCF10A cells were transfected with indicated constructs and cultured for 12 h. Cells were then reseeded at 20% confluency and cultured for another 6 h. EGF (20 ng ml^−1^)was applied after 16 h serum starvation (time 0). Live cells were imaged at indicated time points on a ZEISS LSM 510 DUO microscope (Carl Zeiss MicroImaging) with pinhole set at 1 airy unit using 488 nm excitation and a × 63/1.4 oil objective lens.

### Statistical analysis

All statistical analyses were performed using Excel. All data based on statistical analysis were shown as means±s.d. Statistical significance was analysed by paired Student's *t*-test. All *P* values were two tailed and the level of statistical significance was set at *P*<0.05.

## Additional information

**How to cite this article:** Cao, X. *et al*. A phosphorylation switch controls the spatiotemporal activation of Rho GTPases in directional cell migration. *Nat. Commun.* 6:7721 doi: 10.1038/ncomms8721 (2015).

## Supplementary Material

Supplementary Information Supplementary Figures 1-15 and Supplementary Tables 1-2
