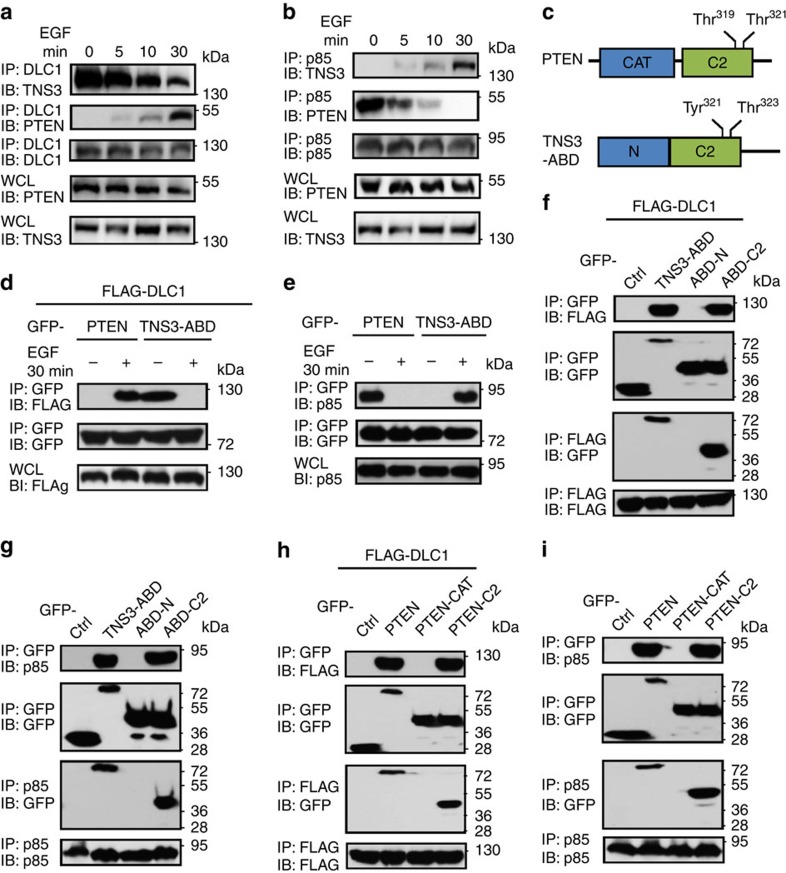# Erratum: A phosphorylation switch controls the spatiotemporal activation of Rho GTPases in directional cell migration

**DOI:** 10.1038/ncomms9320

**Published:** 2015-09-08

**Authors:** Xuan Cao, Tomonori Kaneko, Jenny S. Li, An-Dong Liu, Courtney Voss, Shawn S.C. Li

Nature Communications 6: Article number: 7721 10.1038/ncomms8721 (2015); Published 07132015; Updated 09082015

The image in Fig. 1h of this Article was inadvertently duplicated from panel i. The correct version of the figure appears below.

## Figures and Tables

**Figure 1:**