# Supplementary material for: A phosphorylation switch controls the spatiotemporal activation of Rho GTPases in directional cell migration
Source: Nat Commun. 2015 Jul 13;6:7721. doi: 10.1038/ncomms8721 (PMC4510974; doi:10.1038/ncomms8721)
Supplement: Supplementary Information — Supplementary Figures 1-15 and Supplementary Tables 1-2 [file ncomms8721-s1.pdf]

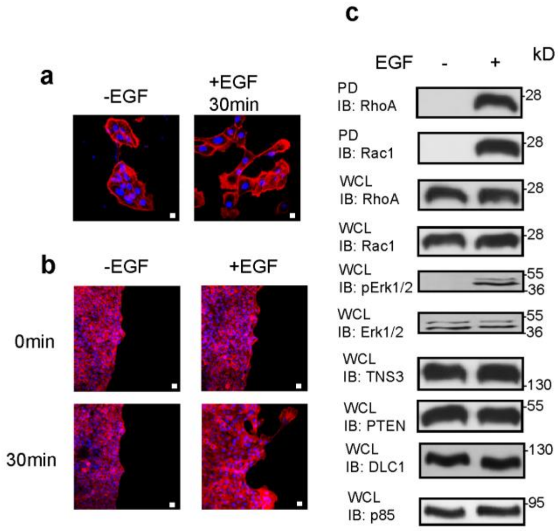

Supplementary Figure 1. Acute EGF treatment promotes mammary cell migration via activation of RhoA and Rac1. (**a**, **b**) Acute EGF treatment (30 min) following serum starvation promotes MCF10A scattering (a) and directional migration (b). Shown are confocal images of cells with DNA (DAPI, blue) and F-actin (rhodamine phalloidin) staining. Scale bar: 10μm. (**c**) Acute EGF treatment (30 min) following serum starvation led to the phosphorylation of Erk1/2 and the activation of RhoA and Rac1, as indicated by the increased levels of RhoA-GTP and Rac1-GTP pulled down, respectively, by the Rhotekin-RBD and PAK-PBD beads. Note that the treatment did not alter the protein levels for TNS3, PTEN, DLC1 or PI3K-p85 in the cells.

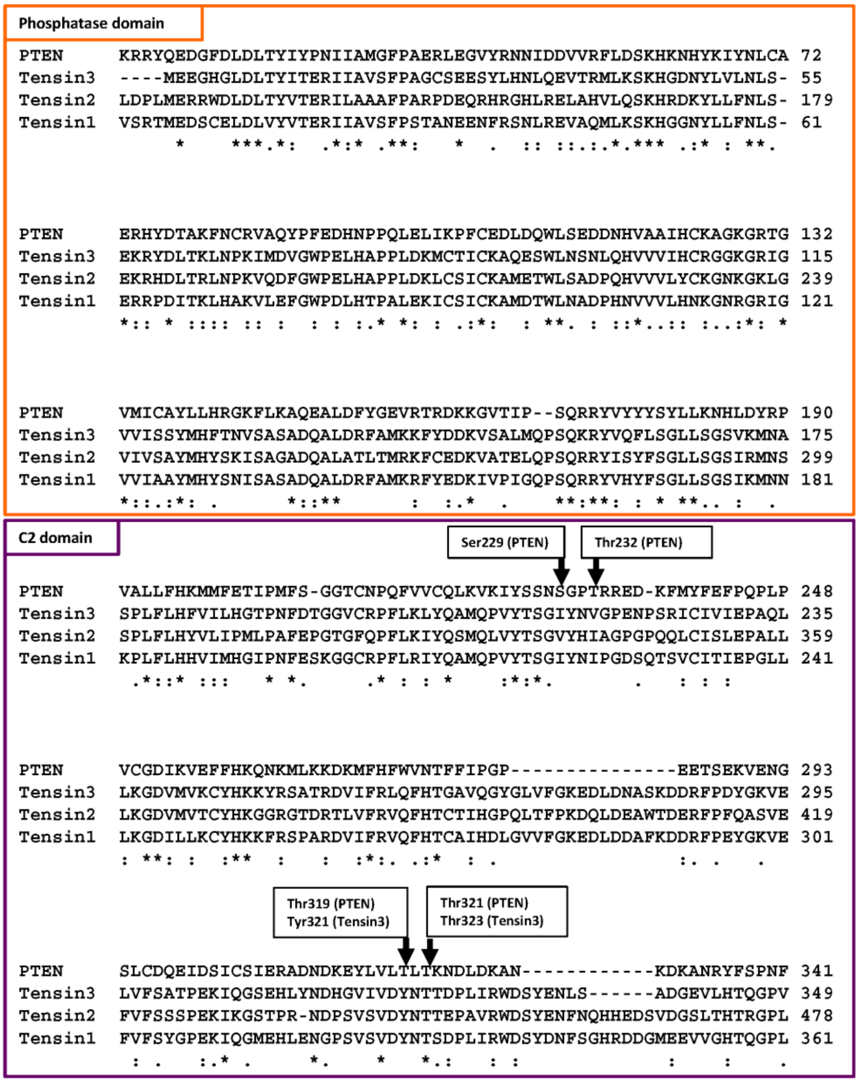

Supplementary Figure 2. Sequence alignment of PTEN against the corresponding regions in Tensin-1 to -3. Note that Thr319 in PTEN is replaced by a Tyr residue in all Tensin C2 domains.

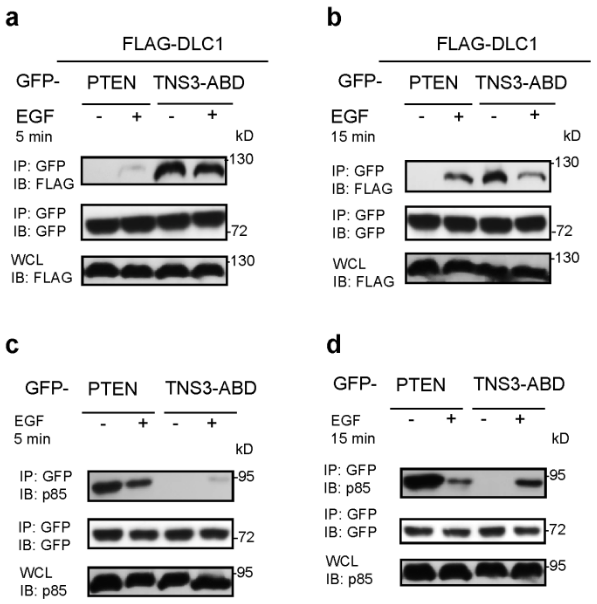

Supplementary Figure 3. EGF signaling triggers binding partner-switch for DLC1, TNS3 (ABD), PTEN and PI3K. (**a**, **b**) EGF treatment of HEK293 cells progressively enhanced PTEN, yet diminished TNS3-ABD binding to DLC1. HEK293 cells were serum-starved and incubated without (-) or with (+) EGF for 5 (a) or 15 (b) min prior to immunoprecipitation (IP) followed by immunoblotting (IB) with the indicated antibodies. PTEN and TNS3-ABD were expressed as GFP fusion and DLC1 with a FLAG-tag to facilitate IP/IB analysis. (**c**, **d**) Acute EGF treatment of HEK293 cells gradually promoted TNS3-ABD, but attenuated PTEN binding to endogenous PI3K-p85. HEK293 cells were subjected to the same treatment as in (a & b) followed by IP/IB with the indicated antibodies.

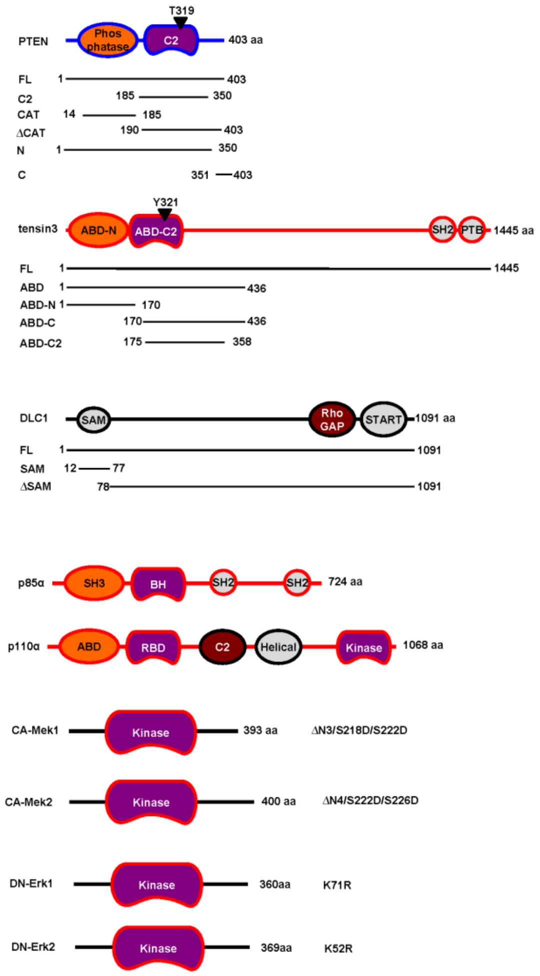

Supplementary Figure 4. A schematic diagram of protein constructs used in this study.

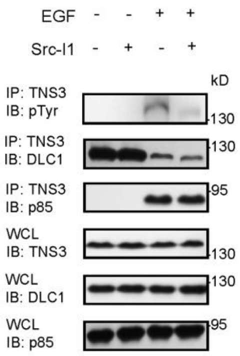

Supplementary Figure 5. Tyrosine phosphorylation of TNS3 had no impact on its binding to DLC1 or PI3K. TNS3 was IP'ed from MCF10A cells that were treated with EGF or/and the Src kinase inhibitor Src-I1 and IB'ed for pTyr, DLC1 and p85, respectively.

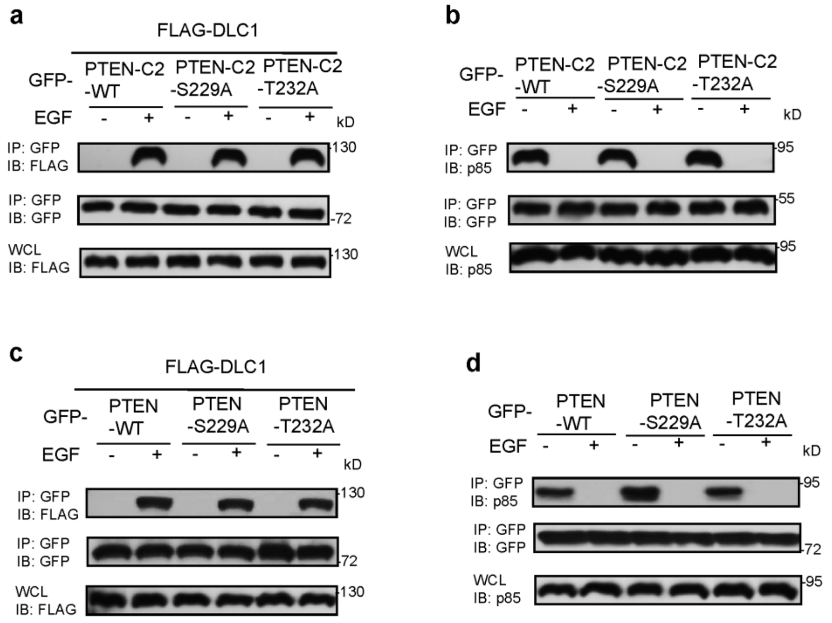

Supplementary Figure 6. The PTEN mutants S229A and T232A exhibited the same affinities as wt PTEN for either DLC1 or PI3K-p85. The experiments were performed under the same conditions as specified in Fig. 3a-d.

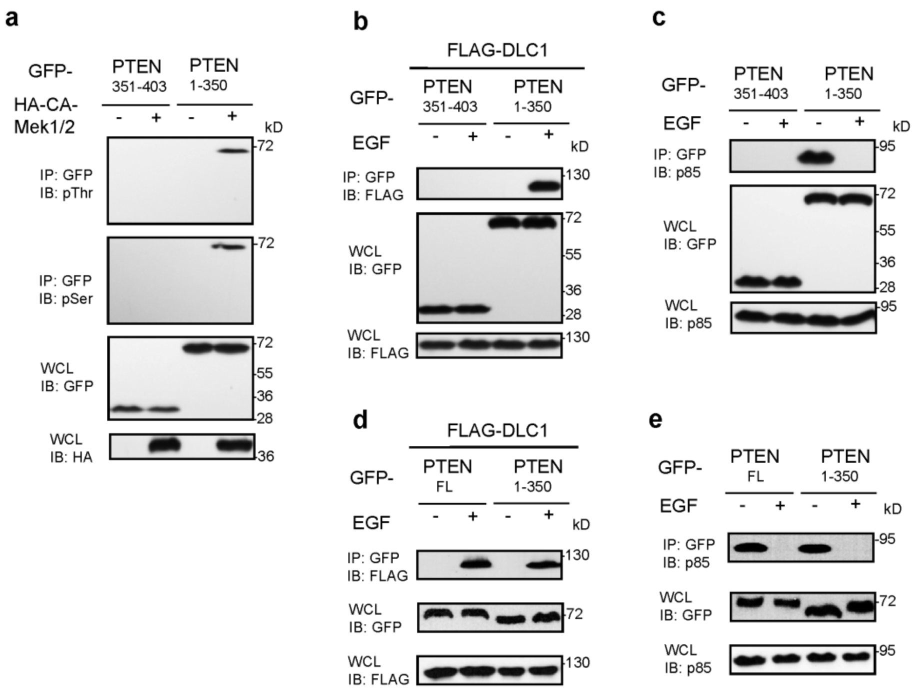

Supplementary Figure 7. The C-terminal region of PTEN is neither phosphorylated by Mek1/2, nor was it involved in binding to DLC1 or PI3K-p85. (**a**) The N- (aa 1-350) and C- (aa 351-403) terminal segments of PTEN were expressed as GFP fusions, respectively, in HEK293 cells that co-expressed (or not) CA-Mek1/2. An anti-GFP IP followed by an anti-pThr or anti-pSer IB showed that the N-terminal, but not the C-terminal segment of PTEN, was phosphorylated. (**b-e**) The same PTEN segments as in (a) were examined for binding to FLAG-DLC1 (**b**, **d**) or PI3K-p85 (**c**, **e**) by IP/IB in serum-starved cells in the absence (-) or presence (+) of EGF.

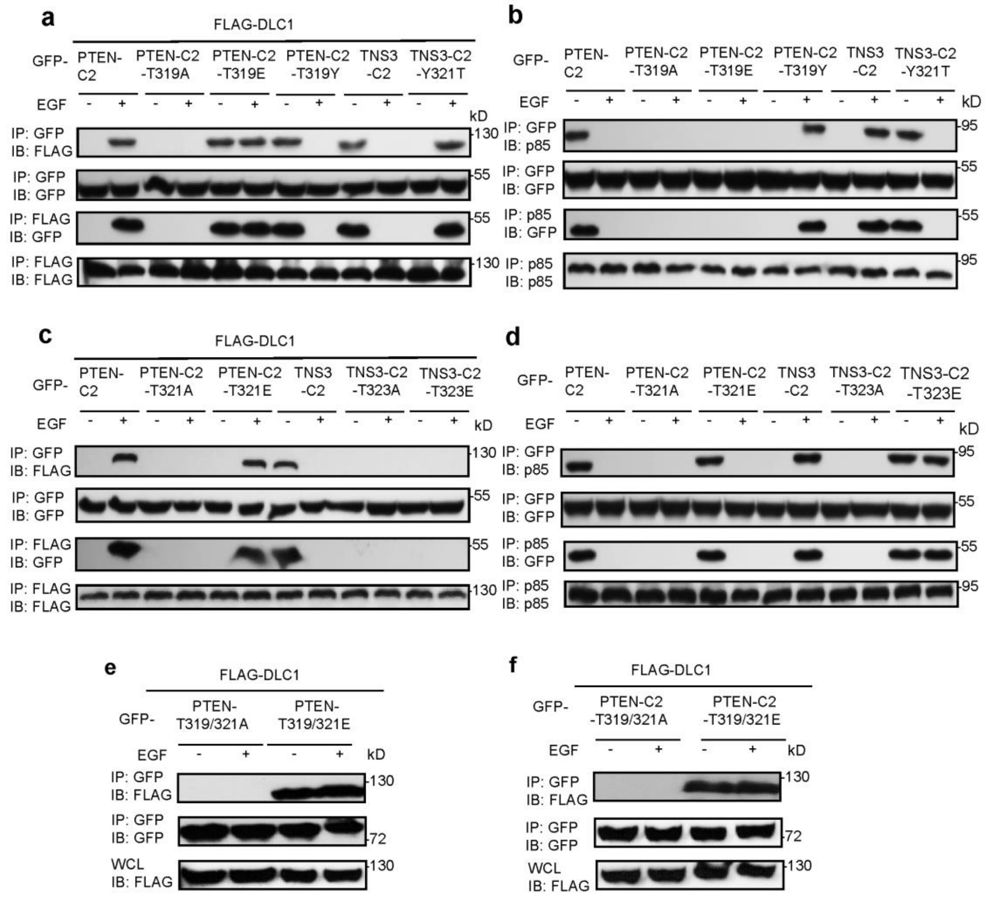

Supplementary Figure 8. The specificities of the PTEN and TNS3 C2 domains are regulated by residues at two conserved positions. (a-d) Thr319 and Thr321 in the PTEN-C2 domain and the equivalent residues Tyr321 and Thr323 in the TNS3-C2 domain regulate their respective binding to DLC1 or PI3K-p85. (**e**, **f**) The PTEN or the C2 domain double mutants T319/321A failed to bind DLC1 whereas the T319/321E mutants bound DLC1 constitutively when they were expressed in HEK293 cells.

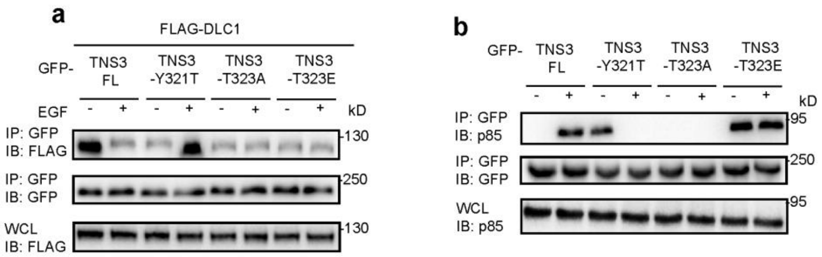

Supplementary Figure 9. Tyr321 and Thr323 regulate binding of full-length TNS3 to DLC1 or PI3K-p85. (**a**) HEK293 cells co-transfected with DLC1 (FLAG-tagged) and TNS3 or a mutant (Y321T, T323A, or T323A, in GFP fusion) were treated with EGF or buffer. IP/IB was carried out to examine DLC1 binding to TNS3 or a mutant. (**b**) Binding of TNS3 or a mutant to endogenous PI3K-p85.

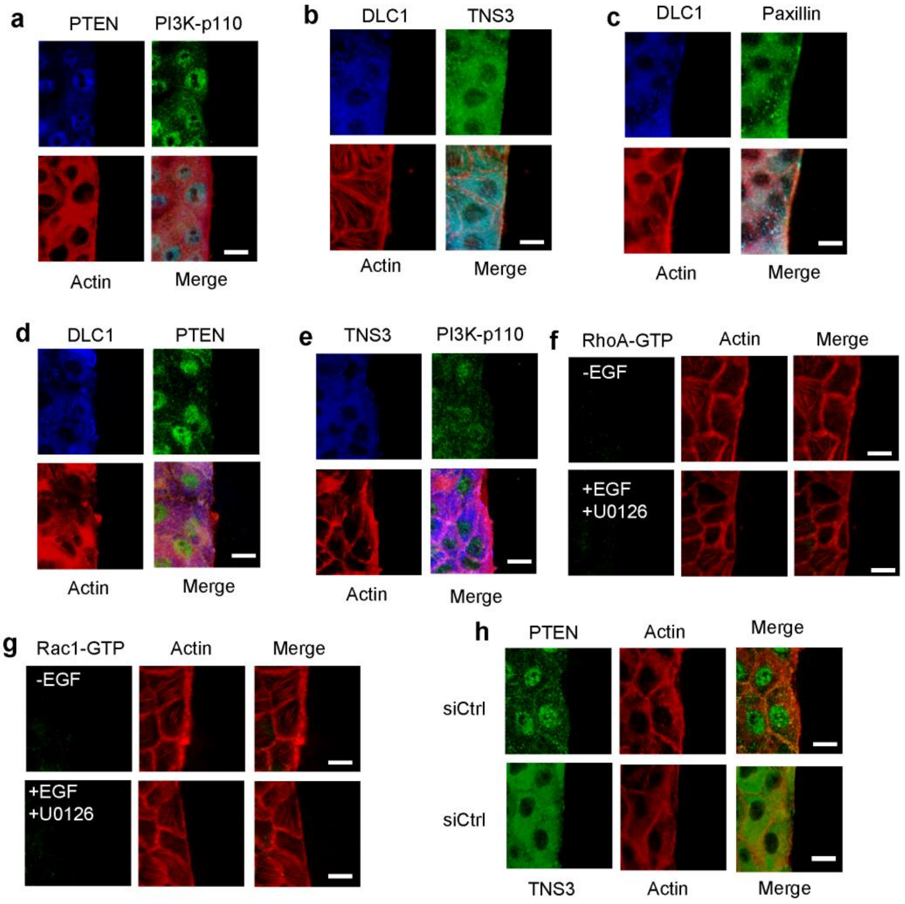

Supplementary Figure 10. Confocal images of cells served as controls for data presented in Figures 5 and 6. (**a-e**) Subcellular localization patterns for DLC1, PI3K-p110, TNS3 and PTEN relative to actin, paxillin or each other in MCF10A cells in the absence of EGF stimulation. (**f**, **g**) Negative RhoA-GTP (f) or Rac1-GTP (g) immunofluorescence (IF) signal without EGF (-) or in the presence of both EGF and U0126. (**h**) Confocal images of MCF10A cells transfected with a control siRNA oligo for PTEN or TNS3. Confocal images shown are representative of four independent experiments. Scale bar: 10μm.

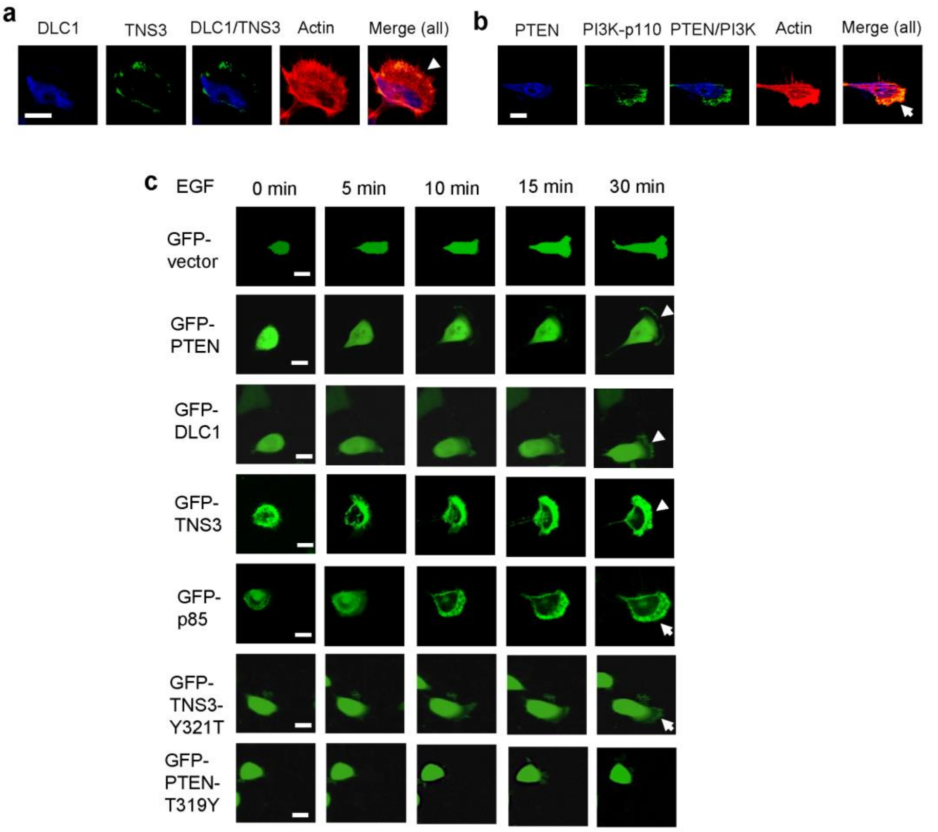

Supplementary Figure 11. Localization of PI3K, PTEN, TNS3 and DLC1 in the migration of single cells in response to EGF stimulation. (**a**) Distinct localization patterns for TNS3 (green) and DLC1 (blue) in EGF-treated MCF10A cells. TNS3, but not DLC1, was found localized in the lamellipodium (stained with rhodamine-phallodin, red). (**b**) PI3K (immunostained in green for the p110 subunit), but not PTEN (blue), localized in the lamellipodium in a migrating cell. (**c**) Dynamic subcellular localization patterns for GFP (control) and GFP-fused PTEN, DLC1, TNS3, p85, TNS3-Y321T and PTEN-T319Y in MCF10A cells during 30 min EGF treatment. Shown are confocal snapshots taken at 0, 5, 10, 15 and 30 min. PTEN and DLC1 were found mainly in the cell body with a weak crescent detected at the cell protrusion at 15 or 30 min. In contrast, TNS3 and PI3K (p85) were found to relocate gradually from the cell body to the leading edge in response to EGF stimulation. The specificity-switching mutant TNS3-Y321T showed the same dynamic localization pattern as PTEN. In contrast, the PTEN-T319Y mutant was found to inhibit the formation of protrusions. Cell protrusions are identified with white arrows. Scale bar: 10μm.

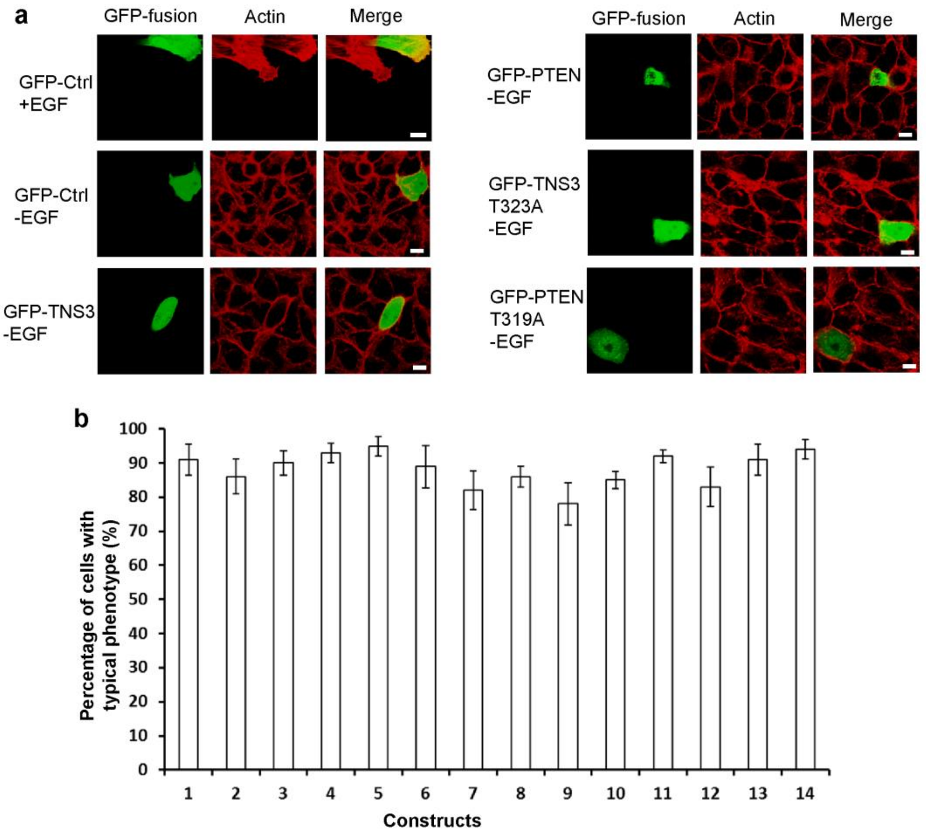

1: GFP-Ctrl (+EGF); **2**: GFP-Ctrl (-EGF); **3**: GFP-TNS3 (-EGF); **4**: GFP-PTEN (-EGF); **5**: GFP-TNS3-T323A (-EGF); **6**: GFP-PTEN-T319A (-EGF); **7**: GFP-PTEN-T319E (-EGF); **8**: GFP-TNS3-T323E (-EGF); **9**: GFP-PTEN-T319Y(-EGF); **10**: GFP-TNS3-Y321T (-EGF); **11**: GFP-PTEN-T319Y (+EGF); **12**: GFP-TNS3-Y321T (+EGF); **13**: HA-CA-Mek1/2 (-EGF); **14**: HA-DN-Erk1/2 (+EGF) .

Supplementary Figure 12. Control confocal images (a) and quantification of data in (a) and those presented in Fig. 6b-e. (**a**) Confocal images of MCF10A cells expressing GFP, GFP-TNS3, GFP-PTEN, GFP-TNS3-T323A, or GFP-PTEN-T319A in the absence (-) or presence (+) of EGF. Scale bar: 10μm. (**b**) Quantitative analysis of transfected cells displaying the characteristic phenotypes as shown in (a) and Fig. 6b-e (images shown are representative of four independent experiments). For each experiment of a construct, 25 transfected cells were randomly selected and examined for the observed phenotype. The percentage of cells with the same phenotype (relative to the total number of analyzed cells) was presented in a bar graph. Numbers shown are mean ± SD (standard deviation), n=4.

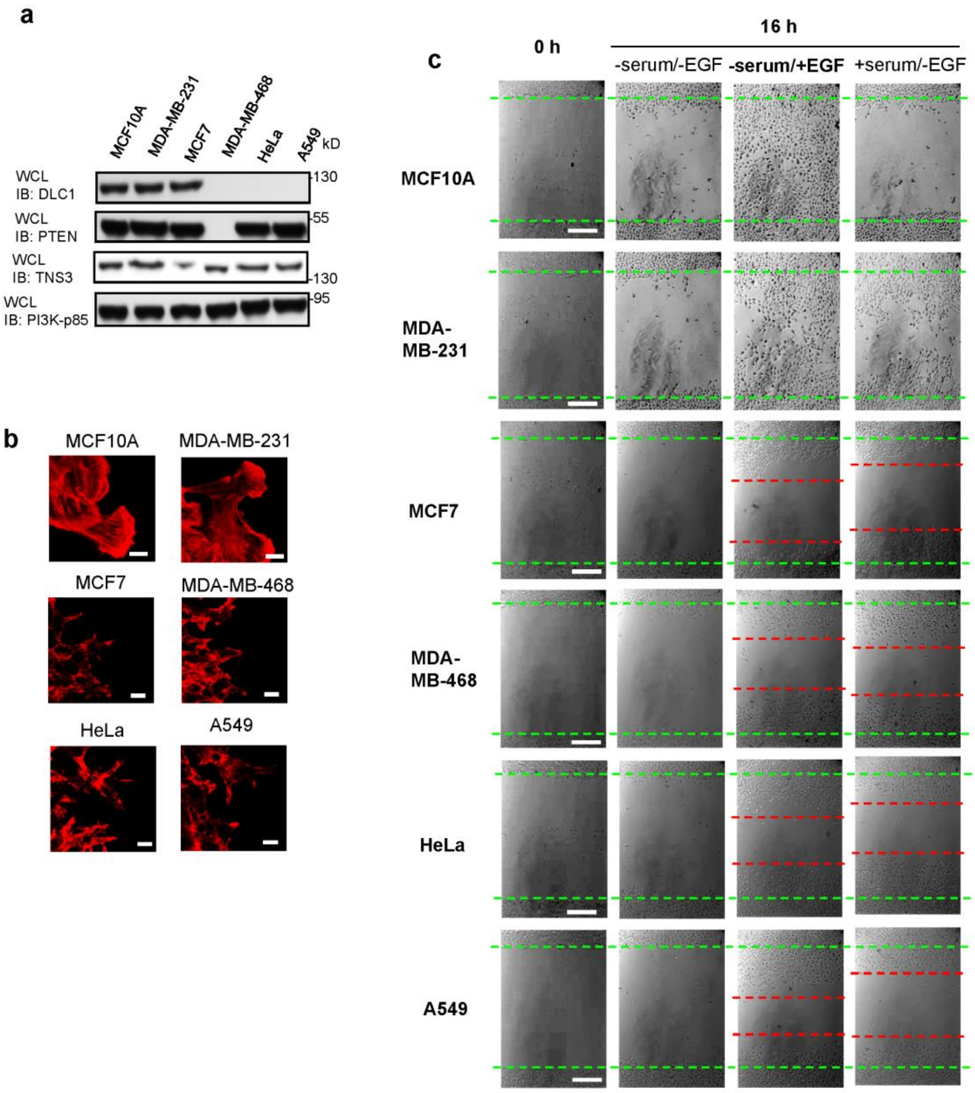

Supplementary Figure 13. Cells missing a component of the "phosphorylation-switch" failed to repair wounds in response to EGF stimulation. (**a**) Western blots to show the levels of DLC1, PTEN, TNS3 and PI3K-p85, respectively, in the indicated cell lines. (**b**) Confocal images to show the formation of lamellipodial protrusiona by the different cells in the presence of EGF. Scale bar: 10μm. (**c**) Phase-contrast images of the indicated cells before (0 h) or 16 h post-scratch. Cells were cultured in a medium lacking both serum and EGF or containing either serum or EGF. Scale bar: 200μm.

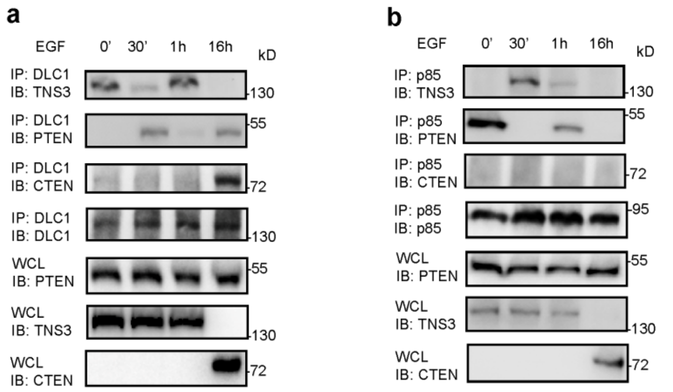

Supplementary Figure 14. Interplay between the "phosphorylation-switch" and the TNS3-Cten "transcriptional switch" during prolonged EGF treatment. Serum-starved MCF10A cells were treated with EGF for 16 hrs. Samples taken at the indicated time points were subjected to immunprecipitation (IP) and immunoblotting (IB) to examine dynamic binding of DLC1 (a) or PI3K-p85 (b) to TNS3, PTEN and CTEN, respectively. Whole cell lysate blots were included to show changes (or lack thereof) for these proteins in response to EGF treatment.

Supplementary Figure 15. Full scans of key Western blots in this study

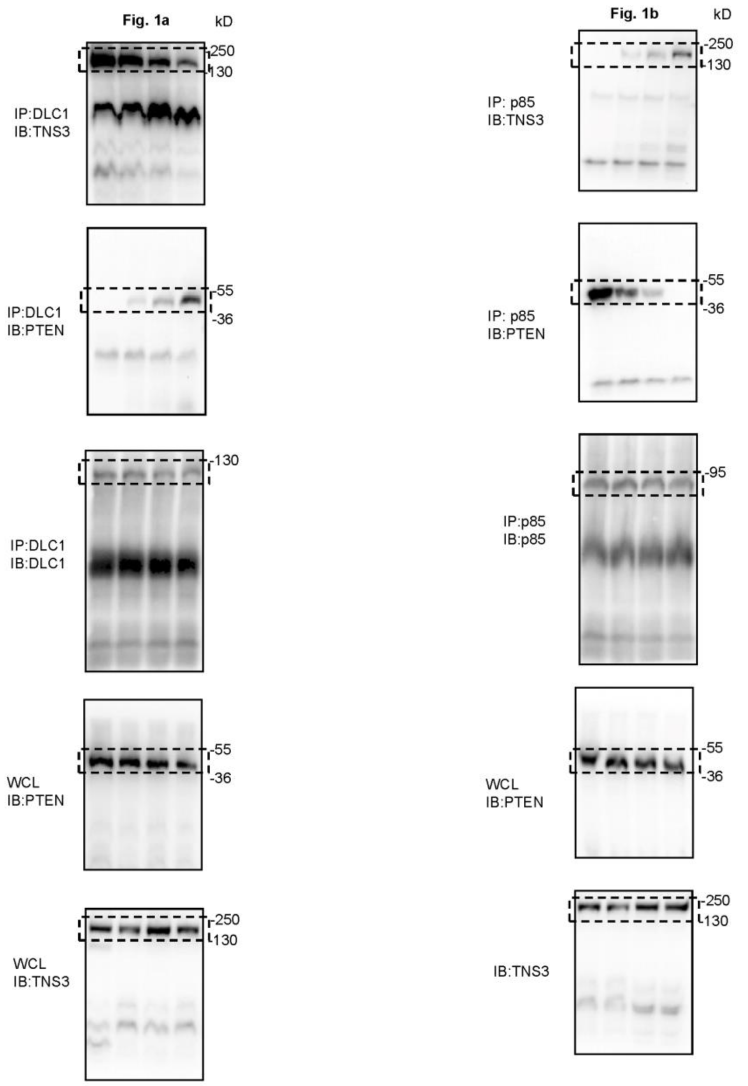

Supplementary Figure 15. Full scans of key Western blots in this study (cont'd)

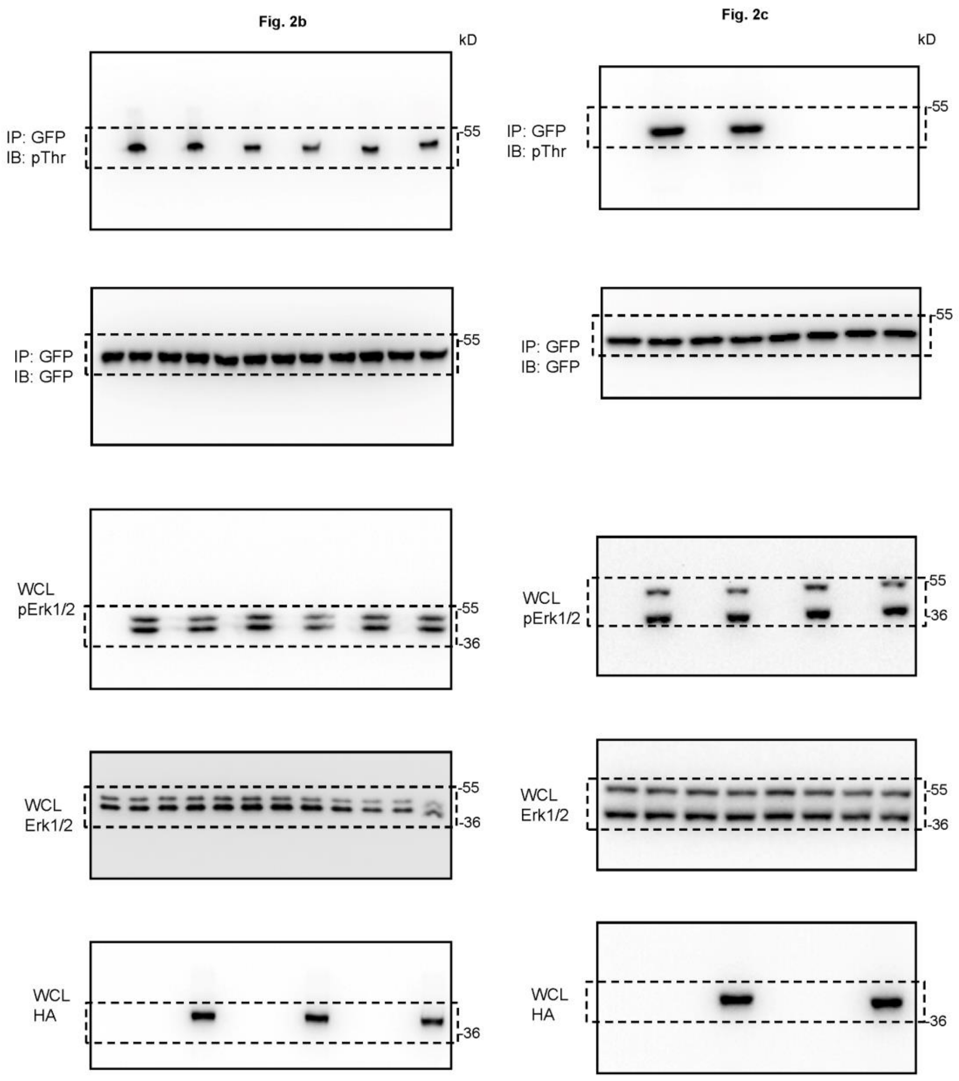

Supplementary Figure 15. Full scans of key Western blots in this study (cont'd)

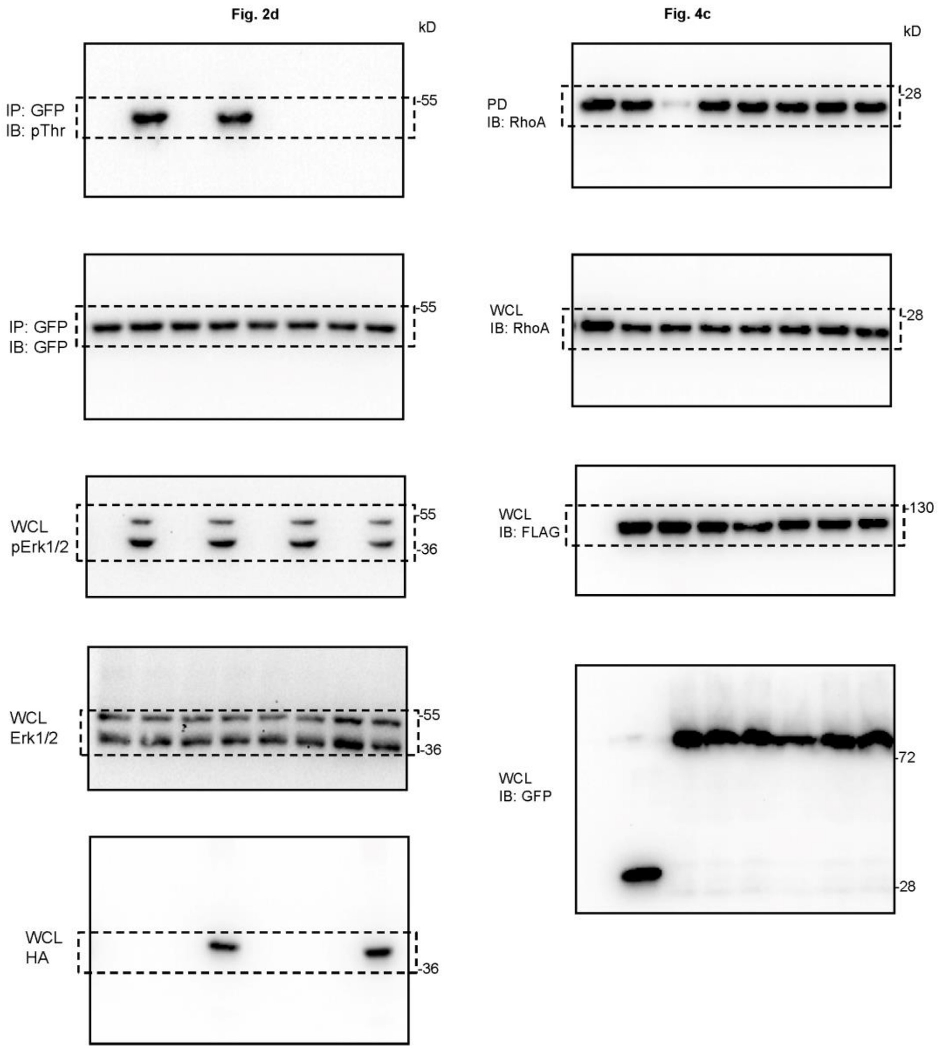

Supplementary Figure 15. Full scans of key Western blots in this study (cont'd)

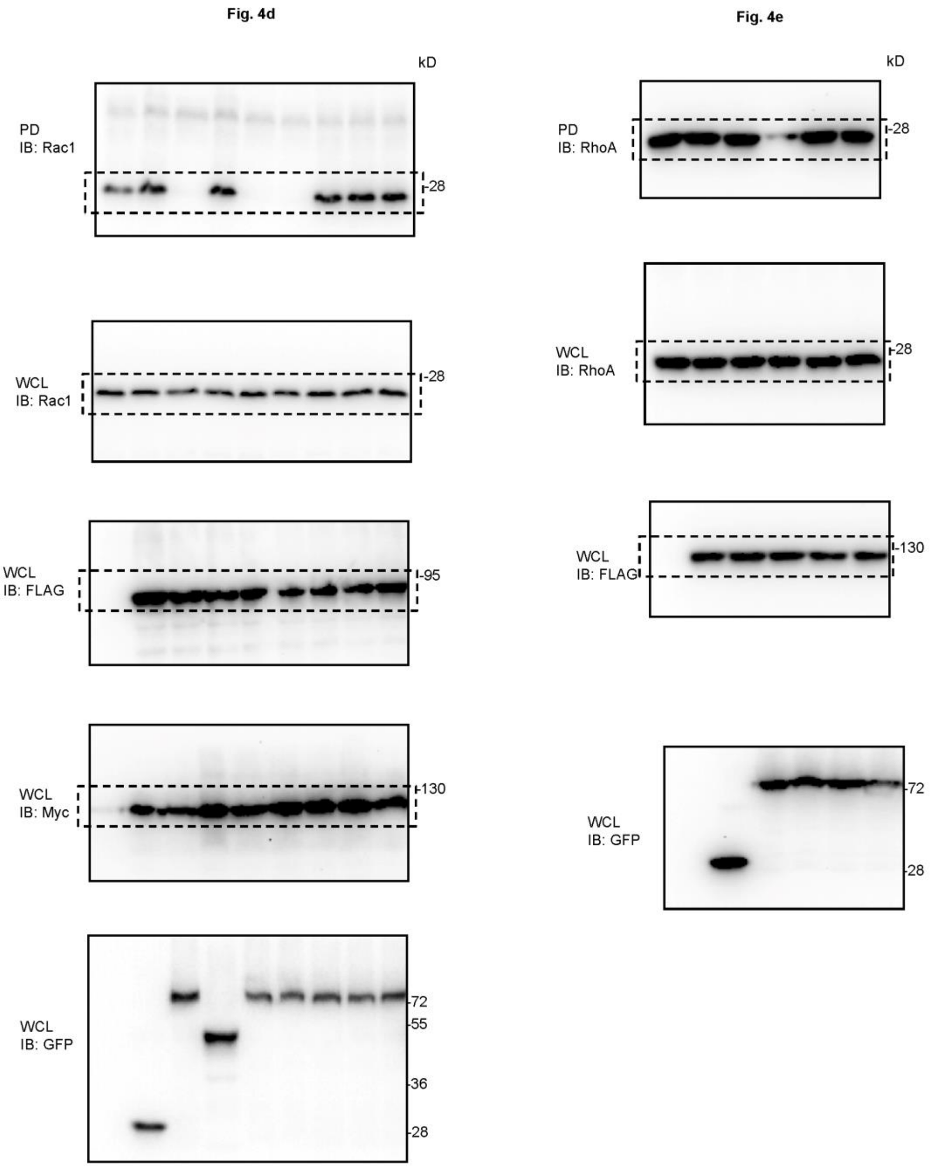

Supplementary Figure 15. Full scans of key Western blots in this study (cont'd)

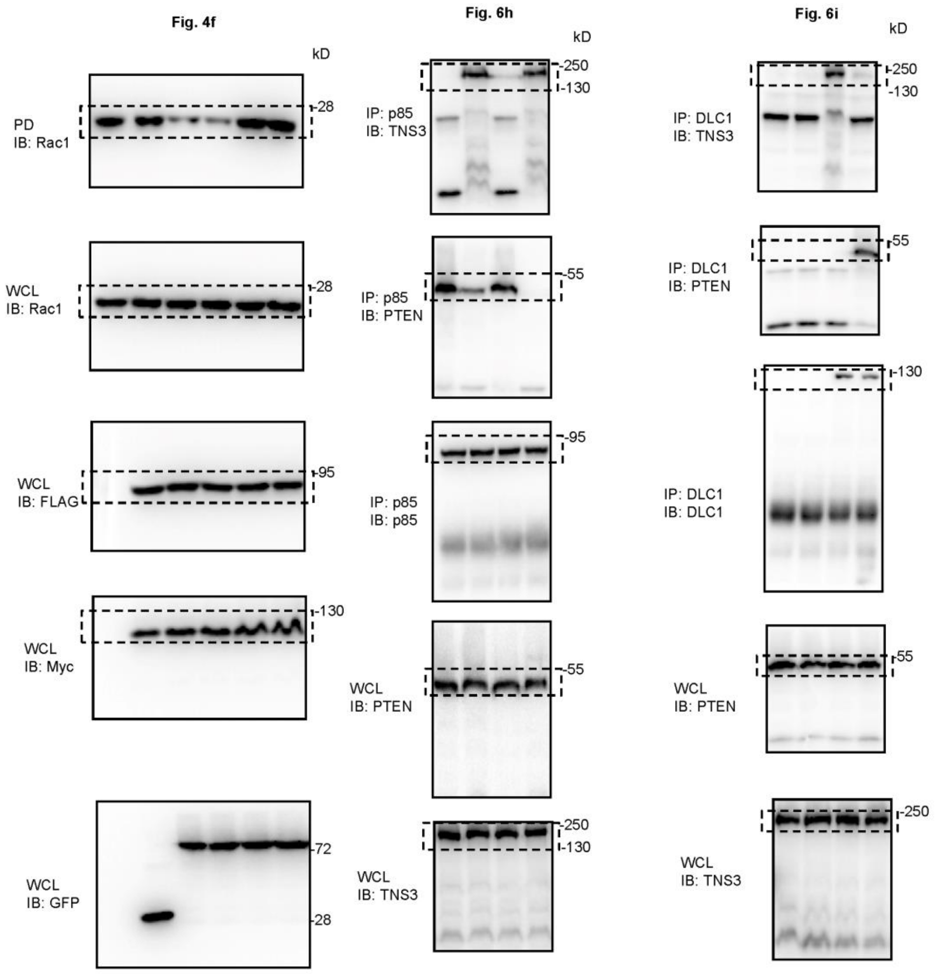

Table 1. Primers for site-specific mutations in PTEN and its C2 domain

(F: forward primer; R: reverse primer)

S229A	F: GATATATTCCTCCAATTCAGGACCC**GCC**CGACGGGAAGACAAGTTC
	R: GAACTTGTCTTCCCGTCG**GGC**GGGTCCTGAATTGGAGGAATATATC
T232A	F: CTCCAATTCAGGACCC**GCC**CGACGGGAAGACAAGTTC
	R: GAACTTGTCTTCCCGTCG**GGC**GGGTCCTGAATTGGAG
T319A	F: GGAATATCTAGTACTT**GCC**TTAACAAAAAATGATC
	R: GATCATTTTTTGTTAA**GGC**AAGTACTAGATATTCC
T319E	F: CAAGGAATATCTAGTACTT**GAG**TTAACAAAAAATGATCTTGAC
	R: GTCAAGATCATTTTTTGTTAA**CTC**AAGTACTAGATATTCCTTG
T319Y	F: GACAAGGAATATCTAGTACTT**TAC**TTAACAAAAAATGATCTTGACAAAGC
	R: GCTTTGTCAAGATCATTTTTTGTTAA**GTA**AAGTACTAGATATTCCTTGTC
T321A	F: ATCTAGTACTTACTTTA**GCC**AAAAATGATCTTGACAAAGC
	R: GCTTTGTCAAGATCATTTTT**GGC**TAAAGTAAGTACTAGAT
T321E	F: ATATCTAGTACTTACTTTA**GAG**AAAAATGATCTTGACAAAGC
	R: GCTTTGTCAAGATCATTTTT**CTC**TAAAGTAAGTACTAGATAT
T319/321A	F: CAAGGAATATCTAGTACTT**GCC**TTA**GCC**AAAAATGATCTTGAC
	R: GTCAAGATCATTTTT**GGC**TAA**GGC**AAGTACTAGATATTCCTTG
T319/321E	F: CAAGGAATATCTAGTACTT**GAG**TTA**GAG**AAAAATGATCTTGAC
	R: GTCAAGATCATTTTT**CTC**TAA**CTC**AAGTACTAGATATTCCTTG

Table 2. Primers for site-specific mutations in TNS3 and its ABD or C2 domain

(F: forward primer; R: reverse primer)

Y321T	F: GGTGTGATTGTGGAC**ACC**AACACAACAGACC
	R: GGTCTGTTGTGTT**GGT**GTCCACAATCACACC
Y321E	F: GGTGTGATTGTGGAC**GAG**AACACAACAGACC
	R: GGTCTGTTGTGTT**CTC**GTCCACAATCACACC
T323A	F: GTGATTGTGGACTACAAC**GCC**ACAGACCCACTGATACG
	R: CGTATCAGTGGGTCTGT**GGC**GTTGTAGTCCACAATCAC
T323E	F: GTGATTGTGGACTACAAC**GAG**ACAGACCCACTGATACG
	R: CGTATCAGTGGGTCTGT**CTC**GTTGTAGTCCACAATCAC
